# Orthogonal Combinatorial Raman Codes Enable Rapid High-Throughput-Out Library Screening of Cell-Targeting Ligands

**DOI:** 10.34133/research.0136

**Published:** 2023-05-17

**Authors:** Yuchen Tang, Xingxing Zheng, Tingjuan Gao

**Affiliations:** China Key Laboratory of Pesticide and Chemical Biology of Ministry of Education, College of Chemistry, Central China Normal University, Wuhan 430079, China.

## Abstract

High-throughput assays play an important role in the fields of drug discovery, genetic analysis, and clinical diagnostics. Although super-capacity coding strategies may facilitate labeling and detecting large numbers of targets in a single assay, practically, the constructed large-capacity codes have to be decoded with complicated procedures or are lack of survivability under the required reaction conditions. This challenge results in either inaccurate or insufficient decoding outputs. Here, we identified chemical-resistant Raman compounds to build a combinatorial coding system for the high-throughput screening of cell-targeting ligands from a focused 8-mer cyclic peptide library. The accurate in situ decoding results proved the signal, synthetic, and functional orthogonality for this Raman coding strategy. The orthogonal Raman codes allowed for a rapid identification of 63 positive hits at one time, evidencing a high-throughput-out capability in the screening process. We anticipate this orthogonal Raman coding strategy being generalized to enable efficient high-throughput-out screening of more useful ligands for cell targeting and drug discovery.

## Introduction

High-throughput assays provide efficient solutions for multiplex genetic analysis, drug discovery, and clinical diagnostics. It allows for simultaneous identification of multiple targets by encoding their recognition counterparts with specific tags [1–6]. Current multiplex assays involve complicated and/or expensive ligand synthesizing, encoding, and decoding processes limiting the capacity of the coding system [5,7,8]. The underlying challenge is to maintain the codes’ survivability and accuracy during the complete lengthy processes. For example, one-bead one-compound (OBOC) combinatorial library screening has been successfully applied to identify functional ligands from thousands to millions of synthetic compounds [4,9–12]. However, the screening efficiency is limited by the traditional slow and costly chemical coding techniques [13]. Because chemical coding strategies utilize molecular monomers as the encoding units [14–16], the codes and the functional ligands are typically the same types of polymer molecules. Although these codes generally survive the synthetic reactions and the subsequent functional tests, codes have to be degraded and eluted from the coding carrier during the decoding process before their sequences are identified by chromatography or mass spectrometry [8,13,14]. Only a few positive beads with the highest binding strengths are able to be selected for obtaining the decoded sequences. Because the time, labor and cost efficiency is considerable low, such types of high-throughput screening are actually “high-throughput in and low-throughput out”.

Compared with the chemical coding strategies, optical coding strategies utilize spectral properties of organic dyes and/or nanoparticles in the dimensions of wavelength, intensity, or their combination [17–19]. Although the codes of positive hits can be identified in situ and rapidly by spectroscopy and/or microscopy, challenges exist for the real high-throughput-out screening in the 3 dimensions of orthogonal factors: (a) whether the codes' spectra remain accurate with the interference from nonspecific signals; (b) whether the codes survive the synthetic reactions producing both the ligands and the codes; and (c) whether the codes stay stable during the functional tests of ligands. In the first dimension of codes' spectral signals, the practical coding capacity is often limited by the overlapping of the coding compounds’ spectra [20–23]. Meanwhile, the background signals from the carriers and ligands may interfere with the codes, too. The utilization of encoding molecular vibrations [24] fulfilled the signal orthogonality and provided a solution to achieve the capacity level of ~10^5^ or higher [25]. Although these molecular vibrational signatures are narrow, distinct, and stable spectral bands [25–27], the synthetic and functional orthogonality of the large-capacity Raman codes remained unexplored. During the processes of synthesizing the functional ligands and analyzing their binding to specific targets, it is challenging for the codes to survive all the reaction conditions, especially in the presence of harsh reagents.

**Fig. 1.**
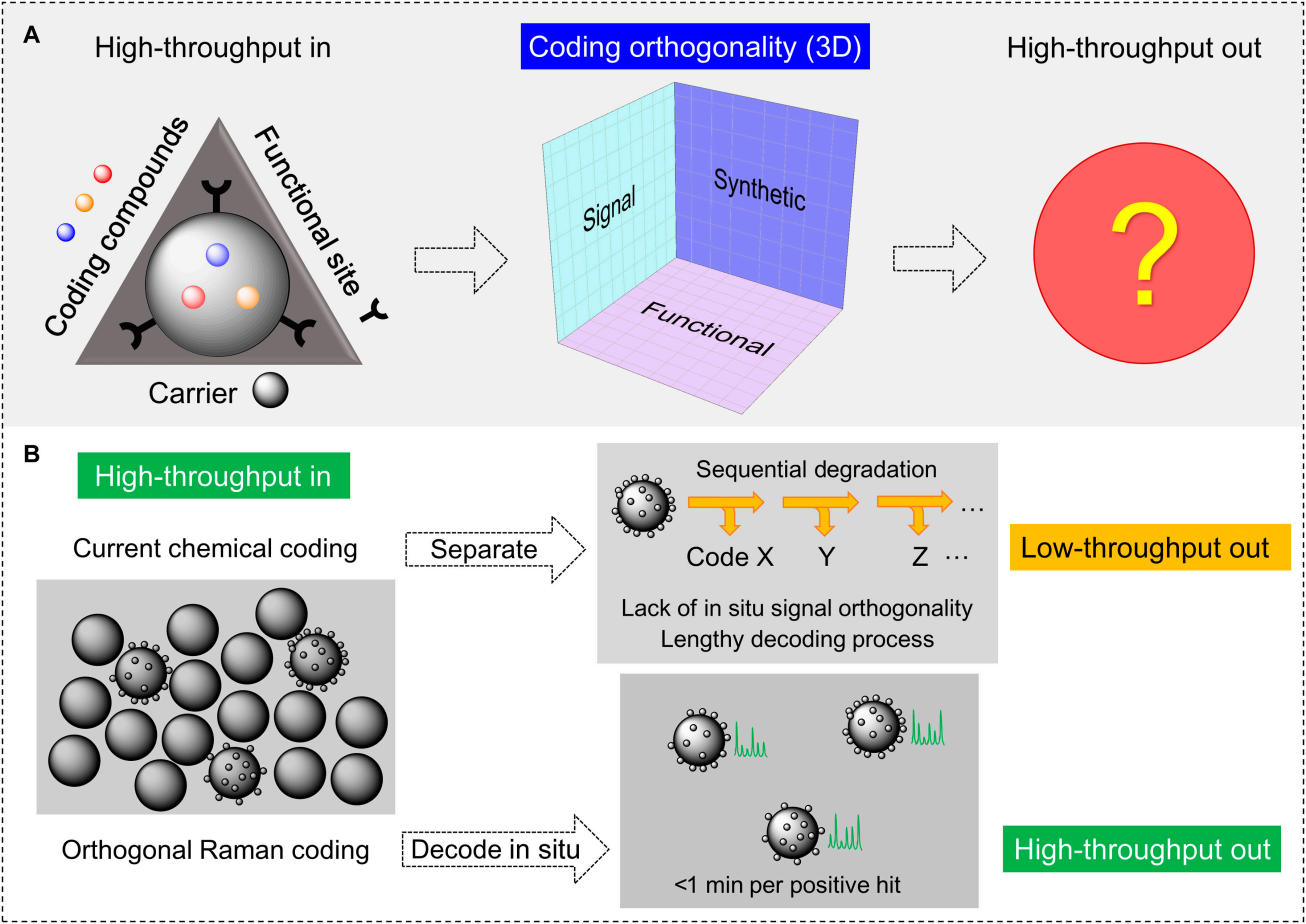
Concept of the coding orthogonality. (A) Schematic of the coding orthogonality in terms of the signal, synthetic, and functional dimensions. (B) Comparison of traditional chemical coding and the new orthogonal Raman coding strategies.

In this work, we developed an orthogonal Raman coding strategy for OBOC library screening to overcome the challenges in the signal, synthetic, and functional dimensions (Fig. 1). A large number of Raman encoding compounds were investigated for their spectral properties and chemical survivability under examplary reaction conditions. Based on the encoding compounds that passed the survivability tests, the sequential combination of their Raman frequency ranges and intensity levels was performed to produce large-capacity codes. Then, we applied the combinatorial Raman coding strategy to simultaneously synthesize the ligands and codes [28]. By screening a focused library of 8-mer cyclic peptides, we rapidly decoded 63 positive sequences in situ from ~12,000 beads. From the statistical distribution of the positive sequences binding specifically with certain types of cancer cells, we discovered multiple peptide ligands with different bioactivities. By applying these orthogonal Raman codes to screen cell-targeting ligands at the high-throughput-out level, we expect the coding strategy to be a promising solution after years of desire for the goal of “high-throughput in and high-throughput out” since the initial demonstration of Raman coding for high-throughput assays (Fig. 1) [29–33].

## Results and Discussion

### Chemical survivability of the Raman encoding compounds

During the process of setting up the codes matching with the peptide sequences on the solid-phase carrier, the codes have to pass the stability test at each step of code synthesis, ligand synthesis, and ligand function generation. The reaction reagents can be categorized into several major types including the Lewis/Brønster acid/base, oxidant/reductant, and Van der Waals interaction (Table 1) in an examplary synthetic route to obtain the combinatorial peptide library and the corresponding Raman codes (Fig. S5). The amide coupling reagent of DIC and Cl-HOBt provides an activation condition for the carboxyl groups on the encoding compounds. Piperidine in DMF is a strong nucleophilic reagent and Brønsted base for the deprotection of Fmoc group. NH_2_OH/imidazole is a reducing agent/base for the orthogonal deprotection of Dde group. TFA is a strong acid for the global deprotection of the amino, hydroxyl, and thiol groups in the final step. DMSO in aqueous solution at pH 6.0 is an oxidizing agent for the dithiol cyclization.

**Table 1.** Representative reaction conditions during OBOC library synthesis and encoding processes.

Reaction conditions	1	2	3	4	5
Property	Activation agent	Base/nucleophile	Base/reductant	Strong acid	Oxidant
Chemicals	Cl-HOBt, DIC in DMF	Piperidine	Imidazole/NH_2_OH	TFA	DMSO in buffer (pH 6.0)
Purpose	Amide coupling	Orthogonal deprotection of Fmoc	Orthogonal deprotection of Dde	Global deprotection	Dithiol cyclization

Experimentally, an encoding compound was coupled to the aminolated resin beads. We investigated the compound’s chemical survivability by measuring the beads’ spectra before and after the treatment of a reagent condition. Since the spectral change was an evidence of the compound degradation under the condition, the unchanged spectrum of the beads indicated the compound,s resistance to the corresponding reagent. The Raman intensity ratio of the compound’s specific peak versus a reference peak (the native polystyrene peak of the beads) was used for the comparison. Based on this method, these 5 types of reagents were tested in an order of their severity levels until each compound was investigated. If the test of a more severe reagent failed, it was not necessay to continue with testing the rest types of reagents, and the outcome was determined as “not pass”. Following this procedure, we investigated 21 Raman compounds and selected the candidates for OBOC library encoding with the “yes” results.

Table 2 lists the chemical survivability result of all the tested encoding compounds. The Raman shifts of these compounds were categorized into 3 major groups from high to low frequencies. Then, the compounds were named with their specific vibrational frequencies. For example, compound I-2295 referred to the first compound in Group I with its specific Raman peak at 2,295 cm^−1^. In the region of Group I (2,300 to 2,200 cm^−1^), Compound I-2295 did not pass the piperidine test, probably due to the instability of aniline amide bond under the nucleophilic condition. Compounds I-2245, I-2225, I-2200, I-2215, and I-2210 survived all the reaction conditions. However, Compound I-2209 failed to pass the initial piperidine test, because the trimethylsilyl group on the diyne was a labile protecting group under basic conditions. In the region of Group II (2,200 to 2,100 cm^−1^), Compounds II-2180 and II-2135 failed to pass the piperidine test, because the extension of alkyne conjugation made the chain more susceptible to the nucleophilic attack. Compounds II-2162 and II-2158 failed to pass the TFA test due to the instability of trimethylsilyl group under acidic conditions. Compound II-2160 successfully passed all the tests because the steric hindrance of triisopropylsilyl substantially improved the resistance to the ambient conditions. Compound II-2156 failed to pass the TFA test, indicating that the aniline amide bond was susceptible to strong acidic conditions. Surprisingly, Compound II-2110 with a straight unprotected phenylacetylene group passed all the test. In the region of Group III (fingerprint region, 1,600 to 1,100 cm^−1^), Compound III-1585 failed to pass the piperidine test, because the long conjugated unsaturated carboxylic acid was susceptible to the nucleophilic attack. Most other compounds (III-1143, III-1140, III-1127, and III-1105) survived all the reaction conditions except for Compound III-1142. The strong TFA condition could result in the hydrolysis of its phenyl ether bond.

**Table 2.**
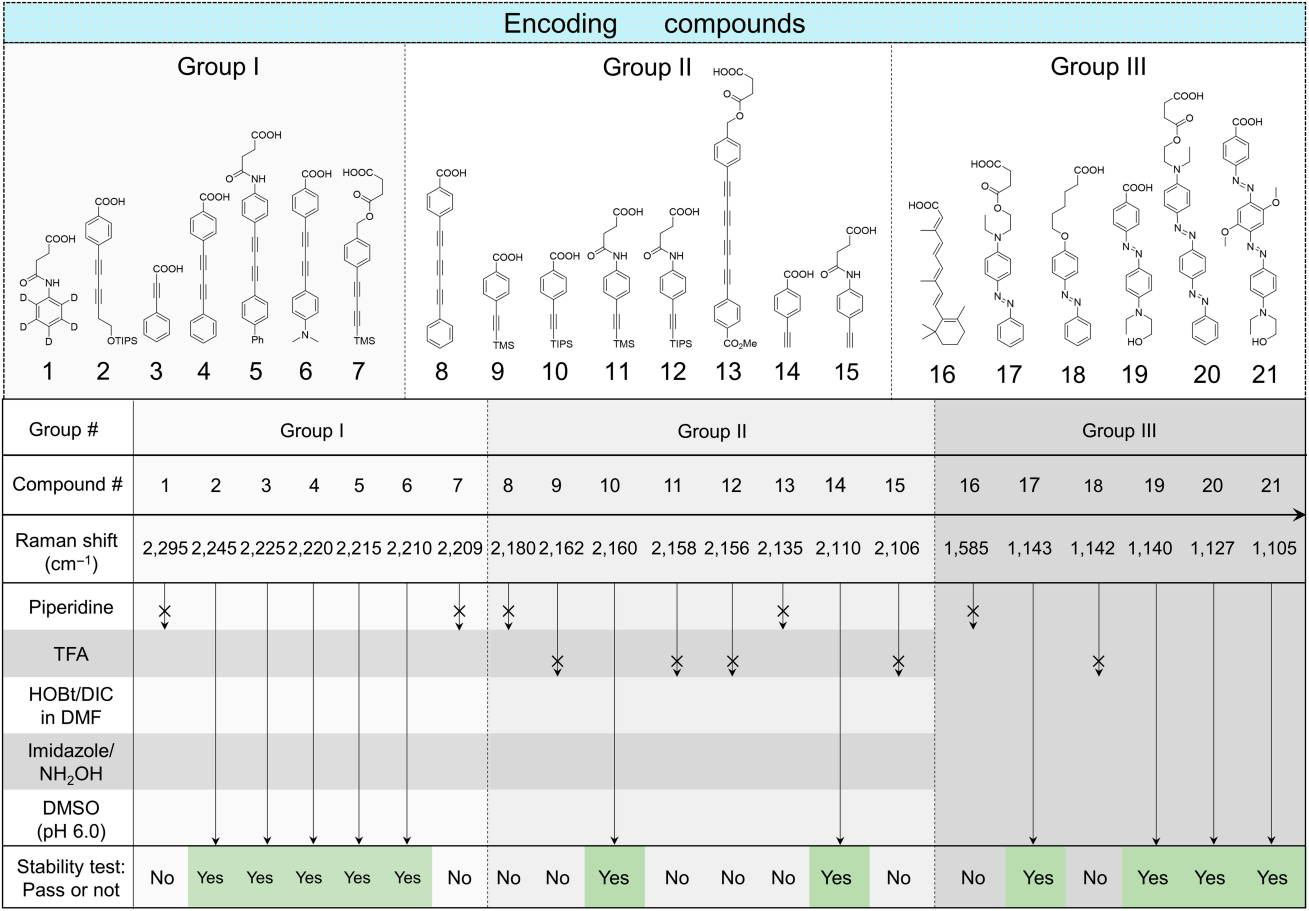
The chemical survivability result of the Raman encoding compounds.

Based on the corresponding structures of the chemical-resistant compounds**,** we selected 2 compounds as a pair from each group of Groups I, II, and III and engineered the combinatorial codes for encoding the OBOC libraries. By tuning their dosages during the synthesis, each frequency region may provide 10 different codes. Then, eventually, we expect to obtain 10^3^ different codes (10 × 10 × 10) when combining the 3 frequency regions together.

### Orthogonality of the combinatorial Raman codes

**Fig. 2.**
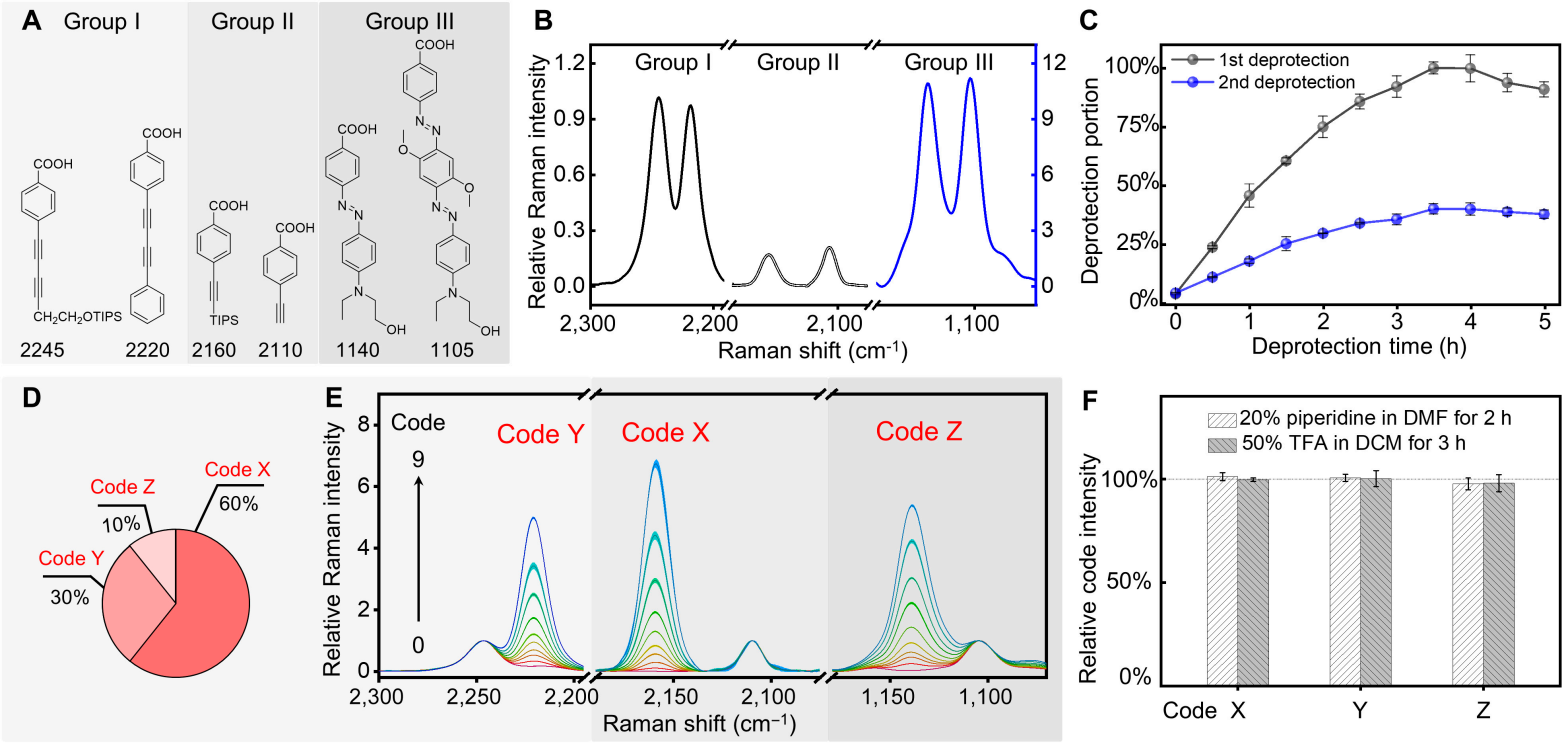
Signal and synthetic orthogonality test of 10^3^ combinatorial Raman codes. (A) Three pairs of encoding compounds in Groups I, II, and III, respectively. (B) Relative Raman intensity levels of the 3 pairs of encoding compounds. (C) Controllable deprotection of Dde groups on the beads. (D) Controllable allocation of amino groups on the beads for synthesizing the codes in the 3 frequency regions. (E) Raman spectra of the combinatorial 10^3^ codes with their standard deviations. (F) Relative Raman intensity levels of codes X, Y, and Z before and after the treatment in piperidine and TFA.

Considering the requirement of distinct frequency differences for the compounds within each pair and the labor/cost efficiency of the compound synthesis, we selected Compounds I-2245 with I-2220, II-2160 with II-2110, and III-1140 with III-1105 (Fig. 2A). Each pair included a reference compound and a coding compound. While the dosage of the coding compound tuned the codes, the Raman intensity of the reference compound was an internal standard to prevent signal fluctuations caused by the synthesis and measurement conditions. To compare the native signal strengths of the 3 compound pairs, we controlled the relative amounts of the 2 compounds within each pair and obtained a similar intensity level for them. After each pair was attached to 100% of the beads’ amino groups, Fig. 2B represents the signal levels of the 3 compound pairs. The intensity level of the Group I pair was approximately 5 times as much as that of the Group II pair, but was approximately ^1^/_10_ of the Group III pair's level.

Due to the different native Raman intensity levels of the 3 compound pairs, the decoding process faced a challenge to accurately collect information from the low-signal regions. Therefore, the relative amounts of the coupling reactions needed to be carefully controlled during the sequential code synthesis for the 3 frequency regions. To achieve similar signal levels in the 3 regions, we investigated the possibility of controlling the deprotection amounts of Dde groups on the beads (Fig. 2C). By carefully adjusting the deprotection time, we were able to control the available portion of the amino groups for synthesizing the codes in the respective frequency regions. Considering the different Raman intensity levels of the 3 compound pairs and the decreasing coupling efficiency during the sequential solid-phase synthesis, the allocation of amino groups were designed in Fig. 2D for synthesizing the codes in the respective frequency regions. We selected the compound pair in Group II to synthesize the codes of the first combinatorial region (named as code X) and utilized the deprotection time of 1.5 h to obtain ~60% of available amino groups for the synthesis. Following the same strategy, we sequentially allocated ~30% and ~10% of amino groups to synthesize code Y and code Z using the Group I and III compound pairs, respectively.

With the controlled allocation of amino groups on the beads, each code series of codes X to Z produced 10 distinct Raman intensity levels when the predesigned amount ratio of the coding compound relative to the reference compound was used (see the Supplementary Materials). After the sequential Dde deprotection and code synthesis processes were combined with the “split and pool” technique, we independently measured the encoded bead batches and confirmed the coding capacity of 10^3^ with the expected accuracy. Figure 2E plots the Raman spectra of the combinatorial 10^3^ codes. Each spectrum was averaged by 5 independent measurements with their standard deviation shown as the shades above and below the curve. The spectra indicate that codes X, Y, and Z presented similar intensity levels for easy decoding identification. If a coding capacity larger than 10^3^ is desired for higher throughput, more combination of the potential encoding compounds can be selected from Fig. 1 or Table 2.

After the 10^3^ codes were obtained, we tested their survivability under different reaction conditions. The encoded beads were treated with the most severe reagents piperidine and TFA among the 5 major types of reagents. Figure 2F indicated that the 10^3^ codes were consistently stable with no observable changes of signal intensities. The results provide us with the confidence in achieving orthogonal high-throughput Raman codes under the real synthetic conditions producing the fully functional ligands in the OBOC libraries.

### High-throughput Raman encoded OBOC library screening

**Fig. 3.**
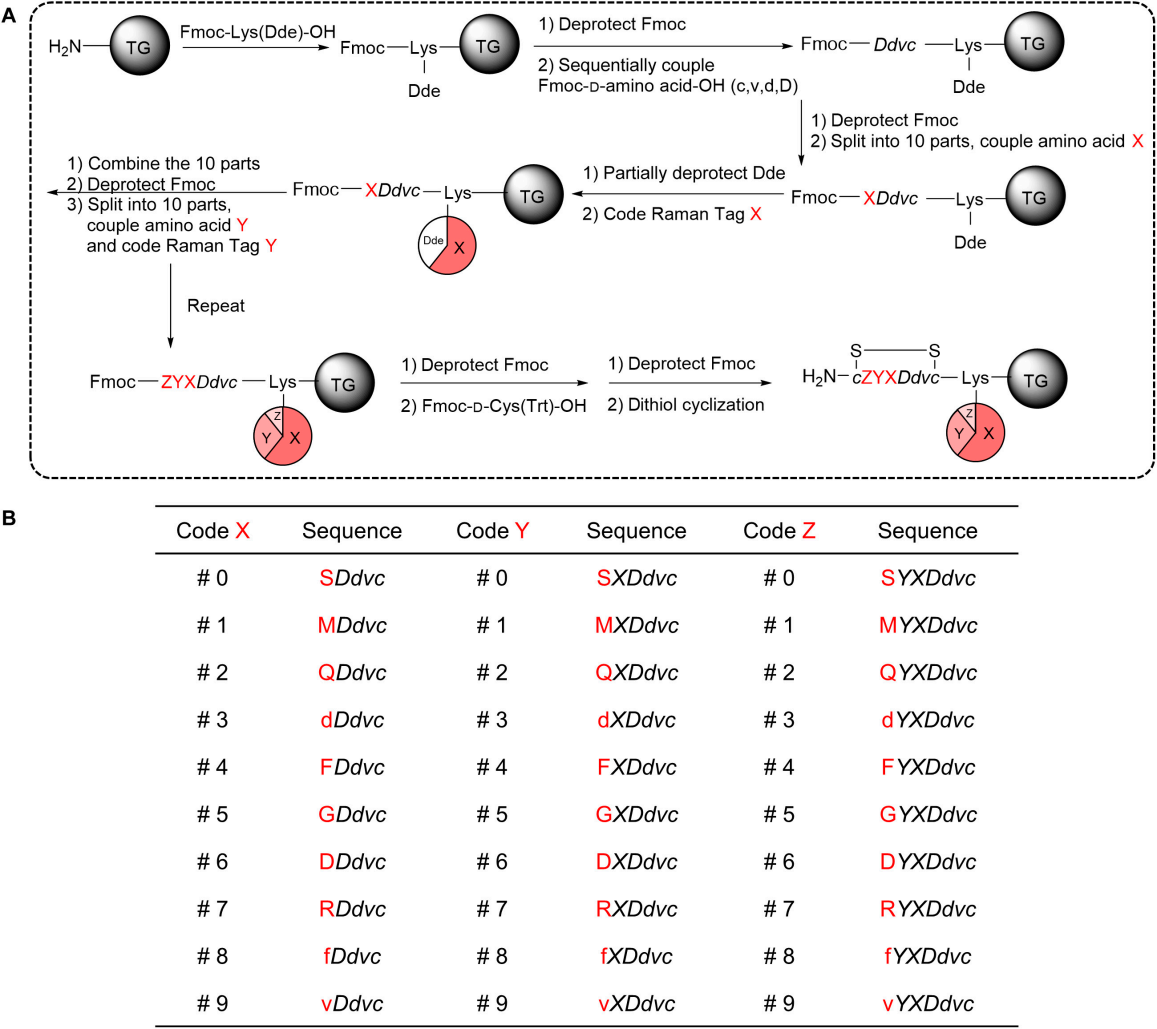
Synthesis and encoding of a focused 8-mer cyclic peptide library. (A) Synthetic route of the library and the 10^3^ combinatorial Raman codes. (B) Design of the combinatorial variations at the 5th, 6th, and 7th amino acids and the corresponding Raman codes. The 10 variations of chiral amino acids are S (l-serine), M (l-methionine), Q (l-glutamine), d (d-aspartic acid), F (l-phenylalanine), G (l-glycine), D (l-aspartic acid), R (l-arginine), f (d-phenylalanine), and v (d-valine).

To prove the high-throughput screening methodology using the combinatorial Raman codes, a focused library of 8-mer cyclic peptides was synthesized and encoded using the scheme shown in Fig. 3A. The 10^3^ combinatorial Raman codes corresponded to the sequences of -*c*ZYX*Ddvc*-, where X, Y, and Z represented the varying amino acids at 5th, 6th, and 7th positions, respectively (Fig. 3B). Among them, code #575 was -*c*GRG*Ddvc*- (LXW7), a previously discovered sequence binding to the α_v_β_3_ integrin on the surface of cancer cells with high affinity [34–36]. During the synthesis of the coding library, we collected a very small portion of beads right after each code was just fabricated. They were measured to provide the calibration of the standard codes (Fig. S6). Then, the spectra of the positive hits after library screening were compared with the standards to decode the peptide sequences.

**Fig. 4.**
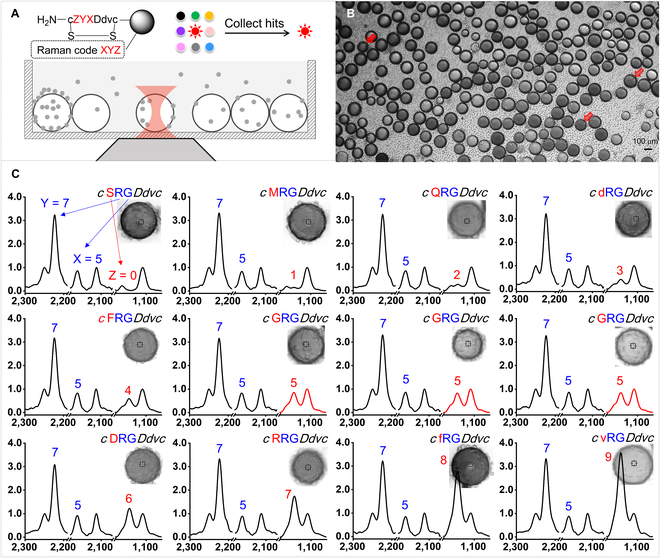
OBOC screening of U-87MG cell binding ligands within a library of 10^3^ peptide sequences. (A) Scheme of in situ rapid decoding using confocal Raman microscopy. (B) Bright-field image of library beads incubated with U-87MG cells. Red arrows highlight positive beads. (C) Examplary spectra of the positive beads and the corresponding peptide sequences.

While this model of OBOC library was designed to discover α_v_β_3_ integrin binding peptides that are potent to cancer cells [34–36], as an examplary demonstration we incubated ~12,000 library beads with U-87MG cells (other types of cancer cells may also be applicable [35]). The experimental scheme (Fig. 4A) followed standard OBOC library screening procedure to identify positive beads with cell-binding ligands [9–12]. Figure 4B represents a bright-field microscopic view of the library beads incubated with U-87MG cells. The red arrows point to a few examplary positive beads. By decoding the positive beads' spectra in situ, we were able to rapidly unveil the positive peptide sequences. Examples of the decoded sequences were demonstrated in Fig. 4C, evidencing that various peptide sequences could bind to U-87MG cells. We compared the positive sequences and discovered that the 5th and 6th amino acids (encoded with code X and code Y) were conservative. However, the 7th amino acid (encoded with code Z) varied among all the 10 alternatives.

**Fig. 5.**
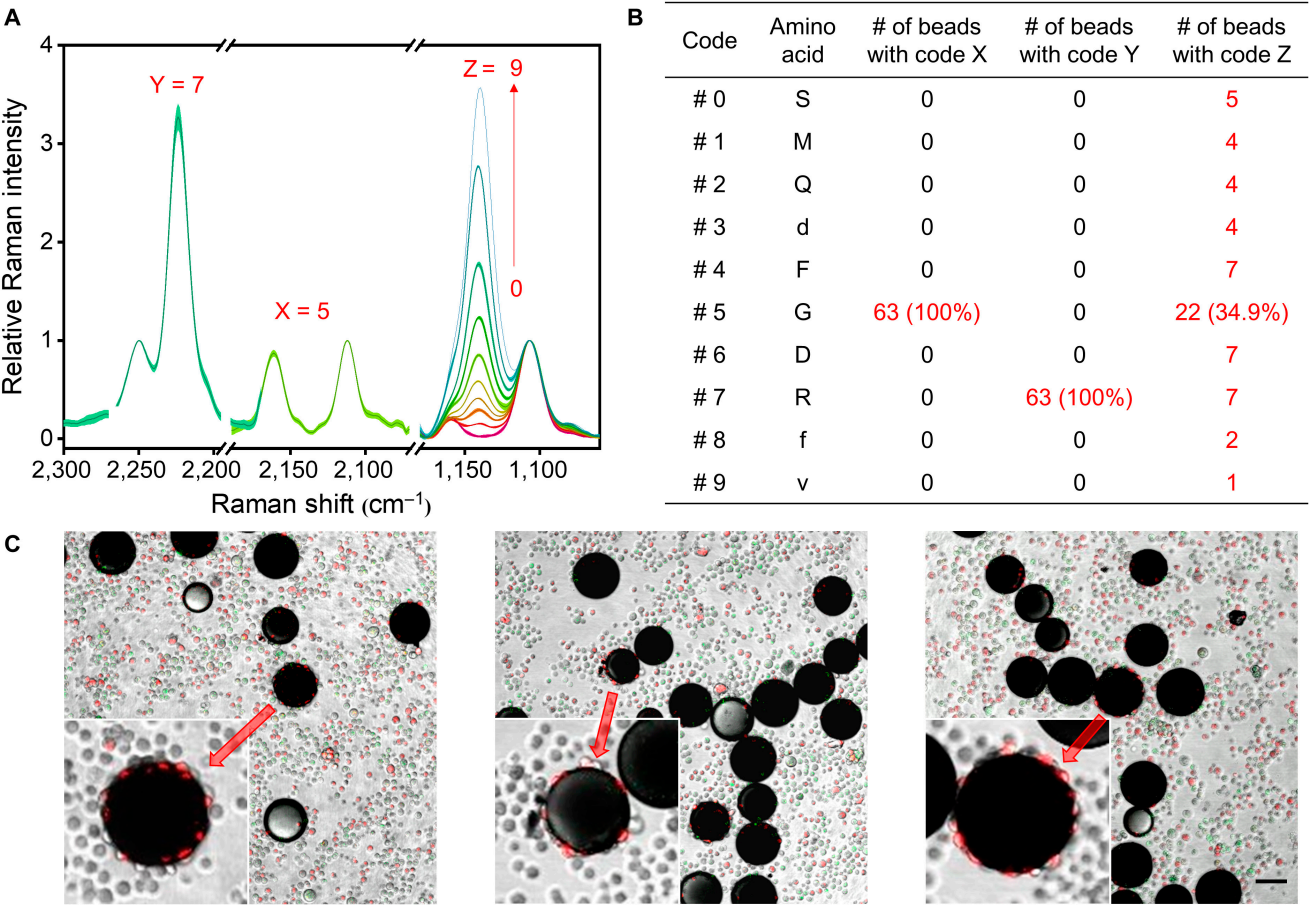
Screening results of the Raman encoded OBOC peptide library. (A) Raman spectra of all the positive beads. (B) Decoding result of the positive beads. (C) Representative 2-focus 3-channel images of the encoded library binding to U-87MG cells, MCF-7 cells, and Hela cells. One focus was at the plane of beads, and the other focus was at the plane of cells. The 3 channels were the bright field, red fluorescence, and green fluorescence. Scale bar, 100 µm.

With all the positive hits identified from ~12,000 beads in the library, we obtained 63 Raman spectra of them. The spectral analysis averaged each same code to a single curve, if multiple beads/spectra corresponded to the same code. Figure 5A illustrates the processed data results. All the spectra were identical at the frequency ranges of codes X and Y. However, they indicated 10 different variations of code Z. Codes X, Y, and Z were determined to be #5, #7, and #0 to #9, respectively. We investigated the distribution of these codes and their corresponding peptide sequences. Fig. 5B lists the number of occurrence for the 5th, 6th, and 7th amino acids (codes X, Y, and Z). The 5th and 6th amino acids had only one possibility. They were G and R, respectively. Different from this, the 7th amino acid could be any of the 10 amino acids, among which the most probable amino acid was G. As the result, all the positive sequences contain -RGD-, and the most probable sequence was -cGRGDdvc- (22 of 63 positive sequences).

The peptide sequence of -cGRGDdvc- (LXW7) was previously discovered to bind specifically to α_v_β_3_ integrin with high affinity [34,35]. α_v_β_3_ plays a key role in the angiogenesis and metastasis of human tumors and is of great interest for advancing targeted therapy and cancer imaging [36]. Based on the structure of LXW7, researchers have performed the sequence optimization by OBOC library screening. However, the screening efficiency was limited by the slow and costly chemical decoding procedure such as Edman degradation combined with high-performance liquid chromatography [13]. Using these traditional decoding techniques, from thousands to millions of sequences in an 8-mer peptide library only a few positive beads with the highest binding strengths were selected for decoding. The rest of many positive beads with less binding strengths had to be discarded or only saved for future decoding. Therefore, the screening test missed substantial useful information that could be obtained from the statistical analysis of all the positive sequences.

Here, we overcame this challenge using the new combinatorial Raman coding methodology. Based on the compatible encoding synthesis and the rapid in situ decoding process (less than 1 min per sequence decoding), the output capacity enabled us to discover the statistical database of all the positive sequences. The methodology may allow researchers to gain deeper understanding of the binding mechanism by comparing the common structures of those positive sequences.

In addition, the combinatorial Raman coding methodology could be combined with multicolor fluorescence imaging to screen ligands targeting different cell lines. Figure 5C demonstrates 3 representative 2-focus 3-channel images of the encoded library binding to the mixed U-87MG cells, MCF-7 cells, and HeLa cells. U-87MG cells and MCF-7 cells were stained with MitoTracker Red and MitoTracker Green, respectively, while HeLa cells were not stained. As a control, the suspended unbound cells in the background clearly demonstrated these 3 types of cells. Then, the overlaid images (bright-field, red-fluorescence, and green-fluorescence channels) identified the cell-targeting morphology of the positive beads and their corresponding cell types. We found that all the positive beads bound with U-87MG cells. Small amount of HeLa cells were also observed on some of the positive beads, indicating their low binding affinity with the ligands. No effective binding was observed for MCF-7 cells. This screening experiment demonstrated an example of expanding additional diversity and throughput in terms of cell targets by exploring applicable target-labeling dyes. Therefore, future applications can be more generalized, as long as the selection of Raman encoding compounds, the target-labeling dyes, and the instrumental excitation/detection frequencies are wisely selected for desired compatibility.

## Conclusion

We developed an orthogonal combinatorial Raman coding methodology with the rapid, reliable, and in situ decoding capability for high-throughput screening of cell-targeting peptide ligands. The chemical-resistant encoding compounds and the constructed 10^3^ combinatorial codes survived 5 types of reaction conditions during the cyclic peptide synthetic process and the subsequent screening assay. With the allocation strategy of partial Dde deprotection and the “split and pool” synthetic technique, the methodology successfully boosted the output capacity of positive sequences without tedious isolation, recovery, and elution steps that are required for the conventional decoding technologies. The orthogonal combinatorial Raman codes provide a desirable solution to analyze sufficient molecular or cellular targets in situ for accurate decoded results with essential simplicity and efficiency. With the chemical compatibility between the library synthesis and the combinatorial Raman coding methodology, we expect the concept of “high throughput” in library screening turns into the reality of “high-throughput in and high-throughput out” in the near future.

## Materials and Methods

### Materials

6-Chloro-1-hydroxybenzotriazole (Cl-HOBt), *N*,*N*′-diisopropylcarbodiimide (DIC), and 9-fluorenyl methoxycarbonyl (Fmoc)-protected chiral amino acids were purchased from GL Biochem (Shanghai, China). *N*,*N*-dimethylformamide (DMF), dimethyl sulfoxide (DMSO), dichloromethane, *N*,*N*-diisopropylethylamine, methanol, *N*-methylpyrrolidone diethyl ether, and trifluoroacetic acid (TFA) were purchased from Sinopharm Chemical Reagent. All other chemical reagents were purchased from Aladdin Bio-Chem Technology (Shanghai, China). TentaGel S NH_2_ resin was purchased from Rapp Polymere GmbH (Tubingen, Germany). Unless otherwise noted, all the materials were used without further purification. U-87MG, MCF-7, and HeLa cells were purchased from Procell Life Science & Technology (Wuhan, China).

The Raman encoding compounds, Raman codes, and Raman encoded peptide library were obtained by Fmoc solid-phase synthesis. Their synthetic details and chemical survivability tests were included in the Supplementary Materials.

### Spectral analysis

Regular Raman spectra of the encoded beads were obtained using a Renishaw inVia reflex spectrometer (Wotton-under-Edge, UK), operating with a 785-nm laser and a thermoelectrically cooled charge-coupled device, coupled to a Leica DM LM microscope (50× air objective; numerical aperture, 0.75). The spectral resolution is 2 cm^−1^. The calibration of the wave number axis was based on measuring the Raman spectrum of a silicon wafer in the static mode. The laser power was 15 mW. When each bead was measured, 5 or 10 frames were collected to average the signals and background noise, where each spectra frame was measured for 2-s exposure. In the library screening experiment, Thermo Fisher DXR Raman microscope was used to image cell binding with a 10× air objective. The spectra of the encoded beads were measured with a 50× air objective and a 785-nm laser. The laser power was 24 mW. The spectra of 10 frames were collected, and each spectra frame was measured for 5-s exposure. All the raw spectra were processed using the Renishaw WiRE software by automatic smoothing and baseline correction when needed. The results were presented by the software of Origin 8.

Nuclear magnetic resonance spectra were recorded on a Bruker AVANCE III 400 MHz spectrophotometer and calibrated using residual undeuterated solvent (^1^H: δ 7.26 for CDCl_3_ and δ 2.50 for DMSO-d6; ^13^C: δ 77.0 for CDCl_3_ and δ 39.50 for DMSO-d6). The high-resolution mass spectra were obtained on an Agilent 6224 time-of-flight mass spectrometer.

### Screening of the Raman encoded peptide library

U-87MG cells were trypsinized with 0.05% trypsin-EDTA from the bottom of a T75 flask and neutralized with the culture medium. Overgrown floating cells were collected, spun down, and resuspended in 4 ml of the culture medium. Two milliliters of the suspension cells in the culture medium were placed into a 35-mm petri dish. The prestored OBOC library beads were added and incubated with U-87MG cells at 37°C with gentle shaking (60 rpm) in a humidified incubator for 1.5 h. After the incubation, the petri dish was placed on the stage of Thermo Fisher DXR Raman microscope. The cell binding was observed under a 10× objective. The Raman spectra of beads were collected under a 50× long-distance objective.

### Multiplex cell line screening with the Raman encoded peptide library

A stock solution of MitoTracker Red and MitoTracker Green was diluted to 10^−4^ M in DMSO. U-87MG and MCF-7 cells were stained in the cell culture with 200 nM MitoTracker Red and 250 nM MitoTracker Green for 20 min, respectively. Hela cells were not stained.

U87-MG, MCF-7, and Hela cells were placed into a 35-mm petri dish. The OBOC library beads were added and incubated in the mixture of U-87MG, MCF-7, and Hela cells. After incubation, the petri dish was placed on the stage of Leica TCS SP8 confocal microscope. The cell binding was observed under a 10× objective at the focus of the beads. The cell morphology and staining were confirmed by the measurement at the focus of unbound suspended cells. The fluorescence images were collected at 488/515 nm and 561/600 nm, respectively.

All other experimental details including the chemical survivability tests, synthesis of the compounds, synthesis of the Raman codes, and the OBOC peptide library are available in the Supplementary Materials.

## Data Availability

The data of this work are available from the corresponding authors upon request.

## Supplementary Materials

Supplementary 1 Synthesis of Raman encoding compounds Chemical survivability tests of Raman encoding compounds Synthesis of the decimal Raman codes Partial allocation of Dde deprotection Synthesis of the combinatorial Raman codes Chemical survivability tests of the combinatorial Raman codes Synthesis of the combinatorial Raman encoded peptide library Figs. S1 to S6 Table S1
