# Supplementary material for: Orthogonal Combinatorial Raman Codes Enable Rapid High-Throughput-Out Library Screening of Cell-Targeting Ligands
Source: Research (Wash D C). 2023 May 17;6:0136. doi: 10.34133/research.0136 (PMC10198463; doi:10.34133/research.0136)
Supplement: Supplementary 1 — Synthesis of Raman encoding compounds Chemical survivability tests of Raman encoding compounds Synthesis of the decimal Raman codes Partial allocation of Dde deprotection Synthesis of the combinatorial Raman codes Chemical survivability tests of the combinatorial Raman codes Synthesis of the combinatorial Raman encoded peptide library Figs. S1 to S6 Table S1 [file research.0136.f1.docx]

**High Throughput in and High Throughput out: Chemical-orthogonal Combinatorial Raman Codes Enable Rapid Library Screening of Cell-targeting Ligands**

Yuchen Tang^*^, Xingxing Zheng, Tingjuan Gao ^*^

China Key Laboratory of Pesticide and Chemical Biology of Ministry of Education, College of Chemistry, Central China Normal University, Wuhan 430079, China

**TABLE OF CONTENTS**

Synthesis of Raman encoding compounds S2

[Synthesis of group I compounds (2100-2200 cm^-1^) S2](#_Toc74916113)

Synthesis of group II compounds(2200-2300 cm-1) S6

[Synthesis of group III compounds(1100-1200 cm-1) S10](#_Toc74916115)

[Chemical survivability tests of Raman encoding compounds S14](#_Toc74916103)

[Synthesis of the decimal Raman codes S15](#_Toc74916102)

[Partial allocation of Dde deprotection S16](#_Toc74916103)

[Synthesis of the combinatorial Raman codes S17](#_Toc74916102)

[Chemical survivability tests of the combinatorial Raman codes S18](#_Toc74916103)

[Synthesis of the combinatorial Raman encoded peptide library S19](#_Toc74916102)

# Synthesis of the Raman encoding compounds

# 4-((trimethylsilyl)ethynyl) benzoic acid (II-2162), 4-ethynylbenzoic acid (II-2110) and retinoic acid (III-1585) were purchased from commercial suppliers and tested without further purification.

## Synthesis of Group Ⅰ compounds (2300 cm^-1^ – 2200 cm^-1^)

To a solution of methyl phenylpropiolate (320 mg, 2.0 mmol) in MeOH/THF (1:1, 20 mL) was added NaOH (400 mg, 10 mmol). The reaction mixture was stirred for 3 h at room temperature and concentrated at reduced pressure. CH_2_Cl_2_ and H_2_O was added to the mixture followed by the addition of aqueous HCl (1 M) to adjust pH to 5. The mixture was extracted with DCM. The organic phase was washed with H_2_O and brine, dried over anhydrous Na_2_SO_4_, filtered and then concentrated at reduced pressure. The residue was purified by column chromatography to afford the product **I-2225** (216 mg, 74.0 %) as a white solid.

To a solution of methyl 4-ethynylbenzoate (320 mg, 2.0 mmol) and phenylacetylene (306 mg, 3.0 mmol) in CHCl_3_/1,4-dioxane (3:1, 8 mL) were added Cu powder (12.7 mg, 0.2 mmol) and TMEDA (88 µL, 0.6 mmol). After the mixture was stirred at 50 ℃ overnight, enough aqueous NH_4_Cl was added. The mixture was extracted three times with EtOAc. The organic phase was washed with H_2_O and brine, dried over anhydrous Na_2_SO_4_, filtered and then concentrated at reduced pressure. The residue was purified by column chromatography to afford the product **S1** (324 mg, 62.3 %) as a white solid.

To a solution of Compound **S1 (**324 mg, 1.24 mmol) in MeOH/THF (1:1, 20 mL) was added NaOH (800 mg, 20 mmol). The reaction mixture was stirred at room temperature for 2 h. The mixture was then concentrated at reduced pressure. CH_2_Cl_2_ and H_2_O was added to the residue, followed by the addition of aqueous HCl (1 M) to adjust pH to 5. The mixture was extracted with CH_2_Cl_2_. The organic phase was washed with H_2_O and brine, dried over anhydrous Na_2_SO_4_, filtered and then concentrated at reduced pressure. The residue was purified by column chromatography to afford the product **I-2220** (287 mg, 94 %) as a white solid. ^1^H NMR (400 MHz, DMSO-*d6*) δ 7.98 (d, *J* = 7.9 Hz, 2H), 7.73 (d, *J* = 7.8 Hz, 2H), 7.66-7.62 (m, 2H), 7.52 (t, *J* = 7.4 Hz, 1H), 7.46 (t, *J* = 7.6 Hz, 2H). ^13^C NMR (101 MHz, DMSO-*d6*) δ 167.0, 133.1, 133.0, 132.0, 130.7, 130.0, 129.4, 125.2, 120.6, 83.6, 81.3, 76.3, 73.7. HRMS (ESI): calcd for C_17_H_9_O_2_^−^ [M-H]^−^ 245.0608, found 245.0603.

Compound **I-2210** was obtained as a light-yellow solid based on the same procedure for preparing Compound **I-2220**. ^1^H NMR (400 MHz, DMSO-*d6*) δ 13.25 (s, 1H), 7.98 (d, *J* = 7.1 Hz, 2H), 7.70 (d, *J* = 7.1 Hz, 2H), 7.45 (d, *J* = 7.7 Hz, 2H), 6.74 (d, *J* = 7.7 Hz, 2H), 3.01 (s, 6H). ESI-MS: calcd for C_19_H_14_NO_2_^-^ [M-H]^-^ 288.1030, found 288.1002.

Compound **S2** was obtained as a light-yellow solid based on the same procedure for preparing **I-2220.** ^1^H NMR (400 MHz, DMSO-*d6*) δ 7.74 (d, *J* = 7.0 Hz, 4H), 7.67 (d, *J* = 7.9 Hz, 2H), 7.52 (t, *J* = 6.9 Hz, 2H), 7.43 (t, *J* = 6.4 Hz, 1H), 7.30 (d, *J* = 8.3 Hz, 2H), 6.59 (d, *J* = 8.3 Hz, 2H), 5.88 (s, 2H).^13^C NMR (101 MHz, DMSO-*d6*) δ 151.2, 141.3, 139.4, 134.4, 133.2, 129.5, 128.5, 127.5, 127.2, 120.6, 114.0, 105.9, 85.4, 81.1, 75.9, 71.8.

To a solution of **S2** (117 mg, 0.40 mmol) in toluene (20 mL) was added succinic anhydride (48 mg, 0.48 mmol) and 4-dimethylaminopyridine (49 mg, 0.40 mmol). The reaction mixture was stirred at 60 ℃ for 3 days. H_2_O was added to the mixture and the mixture was extracted with CH_2_Cl_2_. The organic phase was washed with H_2_O and brine, dried over anhydrous Na_2_SO_4_, filtered and concentrated at reduced pressure. The residue was purified by column chromatography to afford the product **I-2215** (50 mg, 32 %) as a yellow solid. ^1^H NMR (400 MHz, DMSO-*d6*) δ 12.29 (s, 1H), 10.30 (s, 1H), 7.81-7.66 (m, 8H), 7.59 (d, *J* = 8.2 Hz, 2H), 7.52 (t, *J* = 7.4 Hz, 2H), 7.44 (t, *J* = 7.1 Hz, 1H), 2.62 (d, *J* = 5.7 Hz, 2H), 2.59-2.55 (m, 2H). ^13^C NMR (101 MHz, DMSO-*d6*) δ 174.3, 171.1, 141.7, 141.3, 139.3, 133.8, 133.4, 129.6, 128.6, 127.5, 127.2, 120.0, 119.2, 119.2, 114.6, 83.1, 81.9, 75.0, 73.3, 31.6, 29.1. ESI-MS: calcd for C_26_H_18_NO_3_^-^ [M-H]^-^ 392.1292, found 392.1287.

To a solution of (4-ethynylphenyl)methanol (396 mg, 3.0 mmol) and trimethylsilylacetylene (635 µL, 4.5 mmol) in CHCl_3_/1,4-dioxane (3:1, 8 mL) were added Cu powder (19 mg, 0.30 mmol) and TMEDA (136 µL, 0.9 mmol). After the mixture was stirred at 50 ℃ overnight, enough aqueous NH_4_Cl was added. The mixture was extracted three times with EtOAc. The combined organic phase was washed with H_2_O and brine, dried over anhydrous Na_2_SO_4_, filtered and concentrated at reduced pressure. The residue was purified by column chromatography to afford the product **S3** (510 mg, 75 %) as a white solid. ^1^H NMR (400 MHz; CDCl_3_): δ 7.47 (d, *J* = 8.0 Hz, 2H), 7.30 (d, *J* = 8.0 Hz, 2H), 4.67 (s, 2H), 1.95 (br, 1H), 0.23 (s, 9H). ^13^C NMR (101 MHz, CDCl_3_): δ 142.3, 132.9, 126.9, 120.6, 90.8, 87.9, 76.7, 74.3, 64.8, -0.3. HRMS: calcd for C_14_H_15_Si^+^ [M-OH]^+^ 211.0943, found 211.1019.

To a solution of **S3** (43 mg, 0.15 mmol) and succinic anhydride (45 mg, 0.45 mmol) was added N, N-diisopropylethylamine (DIEA, 74 µL, 0.45 mmol) and 4-dimethylaminopyridine (DMAP, 9.2 mg, 0.075 mmol). The reaction mixture was stirred at 40 ℃ overnight. H_2_O was added to the mixture and the mixture was extracted with EtOAc. The organic phase was washed with H_2_O and brine, dried over anhydrous Na_2_SO_4_, filtered and then concentrated at reduced pressure. The residue was purified by column chromatography to afford the product **I-2209** (43 mg, 87 %) as a white solid. ^1^H NMR (400 MHz, DMSO-*d6*) δ 12.30 (s, 1H), 7.60 (d, *J* = 8.1 Hz, 2H), 7.43 (d, *J* = 8.1 Hz, 2H), 5.16 (s, 2H), 2.62 (t, *J* = 6.2 Hz, 2H), 2.54 (t, 2H), 0.25 (s, 9H). ^13^C NMR (101 MHz, DMSO-*d6*) δ 173.9, 172.4, 138.9, 133.1, 128.4, 119.8, 91.9, 88.2, 77.2, 74.4, 65.3, 29.1, 29.1, -0.2. ESI-MS: calcd for C_18_H_19_O_4_Si^-^ [M-H]^-^ 327.1058, found 327.1078.

To a solution of methyl 4-ethynylbenzoate (800 mg, 5.0 mmol) and 3-butyn-1-ol (525 mg, 7.5 mmol) in CHCl_3_/1,4-dioxane (3:1, 24 mL) were added Cu powder (32 mg, 0.50 mmol) and TMEDA (226 µL, 1.5 mmol). After the mixture was stirred at 50 ℃ overnight, enough aqueous NH_4_Cl was added. The mixture was extracted with EtOAc. The organic phase was washed with H_2_O and brine, dried over anhydrous Na_2_SO_4_, filtered and then concentrated at reduced pressure. The residue was purified by column chromatography to afford the product **S4** (320 mg, 28%) as a light-yellow solid.

To a solution of **S4** (114 mg, 0.5 mmol) and triisopropylsilyl chloride (116 mg, 0.6 mmol) in DCM (10 mL) were added imidazole (102 mg, 1.5 mmol) and 4-dimethylaminopyridine (6.1 mg, 0.05 mmol). The mixture was stirred at room temperature for 4 h. H_2_O was added to the mixture and the mixture was extracted with DCM. The organic phase was washed with H_2_O and brine, dried over anhydrous Na_2_SO_4_ and then concentrated at reduced pressure. The residue was subjected to chromatography to produce the TIPS protected ester compound as a pale yellow oil. Then MeOH/THF (1:1, 20 mL) and NaOH (120 mg, 3.0 mmol) were added to the above product. The reaction mixture was stirred at room temperature for 2 h. H_2_O was added to the reaction mixture, followed by the addition of aqueous HCl (1 M) to adjust pH to 5. The mixture was extracted with CH_2_Cl_2_. The combined organic phase was washed with H_2_O and brine, dried over anhydrous Na_2_SO_4_, filtered and then concentrated at reduced pressure. The residue was purified by column chromatography to afford the product **I-2245** (100 mg, 54.1%) as a light-yellow solid. ^1^H NMR (400 MHz, DMSO-*d6*) δ 13.26 (s, 1H), 7.95 (d, *J* = 7.8 Hz, 2H), 7.66 (d, *J* = 7.8 Hz, 2H), 3.85 (t, *J* = 3.8 Hz, 2H), 2.68 (t, *J* = 3.8 Hz, 2H), 1.12 – 1.01 (m, 21H). ^13^C NMR (101 MHz, DMSO-*d6*) δ 167.0, 133.0, 131.7, 130.0, 125.6, 85.7, 77.0, 74.2, 65.8, 61.5, 24.0, 18.2, 11.8. ESI-MS: calcd for C_22_H_29_O_3_Si^-^ [M-H]^-^ 369.1891, found 369.1893.

# To a solution of aniline-d5 (98 mg, 1.0 mmol) and succinic anhydride (120 mg, 1.2 mmol) in acetone (20 mL) was added 4-dimethylaminopyridine (122 mg, 1.0 mmol). The reaction mixture was stirred at 60 ℃ for 3 h. H_2_O was added to the mixture and the mixture was extracted with CH_2_Cl_2_. The organic phase was washed with H_2_O and brine, dried over anhydrous Na_2_SO_4_ and then concentrated at reduced pressure. The residue was purified by column chromatography to afford the product I-2295 (50 mg, 25.3 %) as a light-yellow solid. ^1^H NMR (400 MHz, CDCl_3_) δ 2.70-2.64 (m, 2H), 2.59-2.55 (m, 2H).

# Synthesis of Group II compounds (2200 cm^-1^ – 2100 cm^-1^)

To a solution of 4-ethynylaniline (585 mg, 5.0 mmol) in acetone (20 mL) was added 4-dimethylaminopyridine (610 mg, 5.0 mmol) and succinic anhydride (600 mg, 6.0 mmol). The reaction mixture was stirred at 60 ℃ overnight. H_2_O was added to the mixture and the mixture was extracted with CH_2_Cl_2_. The organic phase was washed with H_2_O and brine, dried over anhydrous Na_2_SO_4_, filtered and then concentrated at reduced pressure. The residue was purified by column chromatography to afford the product **II-2106** (565 mg, 52.1 %) as a light-yellow solid. ^1^H NMR (400 MHz, DMSO-*d6*) δ 12.18 (s, 1H), 10.18 (s, 1H), 7.63 (d, *J* = 8.3 Hz, 2H), 7.43 (d, *J* = 8.3 Hz, 2H), 4.10 (s, 1H), 2.60 (d, *J* = 5.3 Hz, 2H), 2.55 (d, 2H). ^13^C NMR (101 MHz, DMSO-*d6*) δ 174.3, 170.8, 140.3, 132.8, 132.8, 119.1, 119.1, 116.2, 84.1, 80.2, 31.5, 29.1. ESI-MS: calcd for C_12_H_10_NO_3_^-^ [M-H]^-^ 216.0666, found 216.0662.

To a solution of 4-(2-(trimethylsilyl)ethynyl)aniline (567 mg, 3.0 mmol) in acetone (20 mL) was added succinic anhydride (360 mg, 3.6 mmol) and 4-dimethylaminopyridine (366 mg, 3.0 mmol). The reaction mixture was stirred at 60 ℃ overnight. H_2_O was added to the mixture and the mixture was extracted with CH_2_Cl_2_. The organic phase was washed with H_2_O and brine, dried over anhydrous Na_2_SO_4_, filtered and then concentrated at reduced pressure. The residue was purified by column chromatography to afford the product **II-2158** (580 mg, 67 %) as a light-yellow solid. ^1^H NMR (400 MHz, DMSO-*d6*) δ 12.15 (s, 1H), 10.18 (s, 1H), 7.62 (d, *J* = 8.6 Hz, 2H), 7.41 (d, *J* = 8.6 Hz, 2H), 2.60 (d, *J* = 5.4 Hz, 2H), 2.56 (d, *J* = 6.9 Hz, 2H), 0.25 (s, 9H). ^13^C NMR (101 MHz, MeOD) δ 178.1, 174.7, 142.2, 135.2, 122.4, 135.2, 121.4, 108.1, 95.3, 34.2, 31.7, 1.8. ESI-MS: calcd for C_15_H_18_NO_3_Si^-^ [M-H]^-^ 288.1061, found 288.1053.

To a solution of methyl 4--iodobenzoate (1.300 g, 5.0 mmol), palladium (II) acetate (22.4 mg, 0.1 mmol), CuI (9.5 mg, 0.05 mmol), triphenylphosphine (61 mg, 0.2 mmol) and (triisopropylsilyl) acetylene (1.0 mg, 5.5 mmol) were added to 20 mL of triethylamine. The mixture was stirred at N_2_ atmosphere for 2 days and then concentrated at reduced pressure. Ethyl acetate and H_2_O were added, followed by addition of aqueous HCl (1 M) to adjust pH to 5. The mixture was extracted with CH_2_Cl_2_. The combined organic phase was washed with H_2_O and brine, dried over Na_2_SO_4_, filtered and then concentrated at reduced pressure. The residue was subjected to chromatography to give compound **S5** as a light yellow liquid.

To the obtained liquid MeOH/THF (1:1, 20 mL) and NaOH (800 mg, 20 mmol) were added. The reaction mixture was stirred at room temperature for 2 h. Ethyl acetate and H_2_O was then added, followed by the addition of aqueous HCl (1 M) to adjust pH to 5. The mixture was extracted with ethyl acetate. The combined organic phase was washed with H_2_O and brine, dried over anhydrous Na_2_SO_4_ and then concentrated at reduced pressure. The residue was purified by column chromatography to afford the product **II-2160** (906 mg, 66.7 %) as a yellow solid. ^1^H NMR (400 MHz, DMSO-*d6*) δ 13.20 (s, 1H), 7.97 (d, *J* = 6.8 Hz, 2H), 7.60 (d, *J* = 6.8 Hz, 2H), 1.13 (d, *J* = 2.1 Hz, 21H). ^13^C NMR (101 MHz, DMSO-*d6*) δ 167.1, 132.3, 131.2, 130.0, 126.9, 106.7, 93.7, 18.9, 11.1. ESI-MS: calcd for C_18_H_25_O_2_Si^-^ [M-H]^-^ 301.1629, found 301.1630.

Compound **S6** was obtained as a light-yellow solid based on the same procedure for preparing Compound **S5**. To a solution of **S6** (273 mg, 1.0 mmol) in acetone (20 mL) was added succinic anhydride (120 mg, 1.2 mmol) and 4-dimethylaminopyridine (122 mg, 1.0 mmol). The reaction mixture was stirred at 50 ℃ overnight. H_2_O was added to the mixture and the mixture was extracted with CH_2_Cl_2_. The organic phase was washed with H_2_O and brine, dried over anhydrous Na_2_SO_4_ and then concentrated at reduced pressure. The residue was purified by column chromatography to afford the product **II-2156** (213 mg, 57.1 %) as a light-yellow solid. ^1^H NMR (400 MHz, DMSO-*d6*) δ 10.22 (s, 1H), 7.63 (d, *J* = 7.6 Hz, 2H), 7.41 (d, *J* = 7.6 Hz, 2H), 2.98 (t, *J* = 5.5 Hz, 2H), 2.58 (t, *J* = 5.5 Hz, 2H), 1.11 (s, 18H), 1.00 (s, 3H). ^13^C NMR (101 MHz, DMSO-*d6*) δ 170.9, 140.3, 132.8, 131.0, 119.1, 116.8, 112.9, 107.9, 88.9, 19.0, 18.3, 12.5, 11.2. ESI-MS: calcd for C_21_H_30_NO_3_Si^-^ [M-H]^-^ 372.2000, found 372.1991.

Based on the same procedure for preparing Compound **S3**, Compound **S7** was obtained as a light-yellow solid. ^1^H NMR (400 MHz, DMSO-*d6*) δ 7.99 (d, *J* = 8.4 Hz, 2H), 7.74 (d, *J* = 8.4 Hz, 2H), 3.89 (s, 3H), 0.25 (s, 9H). ^13^C NMR (101 MHz, DMSO-*d6*) δ 165.9, 133.4, 130.9, 129.9, 125.2, 93.5, 87.8, 76.7, 76.2, 52.9, -0.2.

To a solution of **S7** (896 mg, 3.5 mmol) in CH_3_CN (20 mL) were added N-bromosuccinimide (748 mg, 4.2 mmol) and AgF (445 mg, 3.5 mmol). The reaction was stirred at room temperature overnight in darkness. Then the mixture was filtered under reduced pressure. The filtrate was poured into H_2_O. The product mixture was extracted with ethyl acetate. The combined organic layer was washed with H_2_O and brine, and dried over anhydrous Na_2_SO_4_. The solution was filtered and concentrated under reduced pressure. The residue was purified by column chromatography to afford the product **S8** (850 mg, 92 %) as a light-yellow solid.

To a solution of **S8** (263 mg, 1.0 mmol) and phenylacetylene (165 µL, 1.5 mmol) in THF (10 mL) were added CuI (9.5 mg, 0.050 mmol), Pd(PPh_3_)_2_Cl_2_ (35 mg, 0.050 mmol) and Et_3_N (277 µL, 2.0 mmol). After the mixture was stirred at room temperature for 1 h, aqueous NH_4_Cl was added. The mixture was extracted with EtOAc. The organic phase was washed with H_2_O and brine, dried over anhydrous Na_2_SO_4_, filtered and then concentrated at reduced pressure. The residue was purified by column chromatography to afford the product **S9** (190 mg, 67 %) as a white solid. ^1^HNMR (400 MHz, DMSO-*d6*) δ 8.02 (d, *J* = 8.3 Hz, 2H), 7.83 (d, *J* = 8.3 Hz, 2H), 7.71 (d, *J* = 7.2 Hz, 2H), 7.58 (t, *J* = 7.4 Hz, 1H), 7.50 (t, *J* = 7.5 Hz, 2H), 3.91 (s, 3H). ^13^C NMR (101 MHz, DMSO-*d6*) δ 165.8, 133.9, 133.9, 133.6, 133.6, 131.3, 131.3, 129.9, 129.9, 129.5, 129.5, 124.6, 119.6, 80.5, 78.5, 76.5, 73.8, 67.9, 66.2, 53.0. ESI-MS: calcd for C_20_H_13_O_2_^+^ [M+H]^+^ 285.0910, found 285.0886.

To a solution of **S9** (142 mg, 0.50 mmol) in MeOH/THF (1:1, 10 mL) was added aqueous NaOH (100 mg, 2.5 mmol). The reaction mixture was stirred at room temperature for 2 h and concentrated at reduced pressure. EtOAc and H_2_O was added, followed by the addition of aqueous HCl (1 M) to adjust pH to 5. The mixture was extracted with EtOAc. The organic phase was washed with H_2_O and brine, dried over anhydrous Na_2_SO_4_, filtered and then concentrated at reduced pressure. The residue was purified by column chromatography to afford the product **II-2180** (125 mg, 93 %) as a white solid. ^1^H NMR (400 MHz, DMSO-*d6*) δ 13.31 (s, 1H), 8.00 (d, *J* = 6.2 Hz, 2H), 7.80 (d, *J* = 6.2 Hz, 2H), 7.70 (d, *J* = 6.6 Hz, 2H), 7.56 (d, *J* = 5.9 Hz, 1H), 7.50 (d, *J* = 6.6 Hz, 2H). ^13^C NMR (101 MHz, DMSO-*d6*) δ 166.9, 133.7, 133.7, 133.6, 133.6, 132.7, 131.3, 130.1, 130.1, 129.5, 129.5, 124.1, 119.7, 80.4, 78.7, 76.2, 73.9, 67.7, 66.3. ESI-MS: calcd for C_19_H_9_O_2_^-^ [M-H]^-^ 269.0608, found 269.0599.

To a solution of **S3** (56.8 mg, 0.25 mmol) and **S7** (51.2 mg, 0.20 mmol) in CH_2_Cl_2_/MeOH (1:1, 2 mL) was added K_2_CO_3_ (276 mg, 2.0 mmol). The mixture was stirred at room temperature for 2 h and filtered at reduced pressure. Then H_2_O was added to the filtrate and the mixture was extracted with CH_2_Cl_2_. The organic phase was washed with H_2_O and brine, dried over anhydrous Na_2_SO_4_, filtered and then concentrated at reduced pressure. The residue was used directly for the next step.

To the solution of the above mixture in CHCl_3_-1,4-dioxane (3:1, 24 mL) were added Cu powder (1.3 mg, 0.02 mmol) and TMEDA (91 µL, 0.60 mmol). The mixture was stirred at 50 ℃ for 2 days and concentrated at reduced pressure. Aqueous NH_4_Cl was then added to the solution and the mixture was extracted with ethyl acetate. The combined organic phase was washed with H_2_O and brine, dried over anhydrous Na_2_SO_4_ and filtered and then concentrated at reduced pressure. The residue was purified by column chromatography to afford the product **S10** (24.5 mg, 36 %) as a light-yellow solid.

To a solution of **S10** (5.0 mg, 0.015 mmol) and succinic anhydride (4.5 mg, 0.045 mmol) in CH_2_Cl_2_-DMF (10:1, 1.1 mL) were added Et_3_N (8.3 µL, 0.060 mmol) and 4-dimethylaminopyridine (0.9 mg, 0.0074 mmol). The mixture was stirred at 40 ℃ for 6 h and concentrated at reduced pressure. H_2_O was added to the mixture and the mixture was extracted with ethyl acetate. The organic phase was washed with H_2_O and brine, dried over anhydrous Na_2_SO_4_, filtered and then concentrated at reduced pressure. The residue was purified by column chromatography to afford the product **II-2135** (3.0 mg, 46 %) as a yellow solid. ^1^H NMR (400 MHz, DMSO) δ 12.27 (s, 1H), 8.00 (t, 2H), 7.83 (d, *J* = 4.3 Hz, 2H), 7.70 (d, *J* = 5.2 Hz, 2H), 7.46 (d, *J* = 8.4 Hz, 2H), 5.18 (s, 2H), 3.89 (s, 3H), 2.92 - 2.76 (m, 2H), 2.62 (d, *J* = 6.5 Hz, 2H). ^13^C NMR (101 MHz, DMSO) δ 173.9, 172.4, 165.8, 140.0, 134.2, 134.2, 134.0, 134.0, 131.5, 129.9, 129.9, 128.4, 128.4, 124.0, 118.5, 79.4, 77.9, 76.2, 74.0, 68.2, 66.9, 65.2, 64.8, 63.7, 53.0, 29.1, 29.1. ESI-MS: calcd for C_27_H_17_O_6_^-^ [M-H]^-^ 437.1031, found 437.1004.

## Synthesis of group ⅠII compounds (1200 cm^-1^ – 1100cm^-1^)

To a solution of N-ethyl-N-hydroxyethylaniline (825 mg, 5.0 mmol) and succinic anhydride (750 mg, 7.5 mmol) in 20 mL of CH_2_Cl_2_ was added 4-dimethylaminopyridine (DMAP, 610 mg, 5.0 mmol) and Et_3_N (1.38 mL, 10 mmol). The reaction mixture was stirred at room temperature for 12 h. H_2_O was added to the mixture and the mixture was extracted with ethyl acetate. The organic phase was washed with H_2_O and brine, dried over anhydrous Na_2_SO_4_, filtered and then concentrated at reduced pressure. The residue was purified by column chromatography to afford the product **S11** (1.27 g, 95%) as a white solid. ^1^H NMR (400 MHz, CDCl_3_) δ 7.26 - 7.21 (m, 2H), 6.87 - 6.53 (m, 3H), 4.25 (q, *J* = 6.0, 5.6 Hz, 2H), 3.55 (t, *J* = 6.4 Hz, 2H), 3.40 (q, *J* = 6.9 Hz, 2H), 2.68 - 2.60 (m, 4H), 1.16 (t, *J* = 6.4 Hz, 3H). ^13^C NMR (101 MHz, CDCl_3_) δ 178.2, 177.6, 147.4, 129.3, 116.3, 112.0, 62.1, 48.7, 45.2, 28.8, 20.8, 12.1. HRMS (ESI): calcd for C_14_H_18_NO_4_^-^ [M-H]^-^ 264.1241, found 264.1237.

Aniline (46.5 mg, 0.50 mmol) in 2 mL of ethanol was cooled to 0℃. Pre-cooled aqueous solution of potassium nitrite (85 mg, 1.0 mmol) and sulfuric acid (147 mg, 0.15 mmol) were added to the mixture. The mixture was stirred at 0°C for 1 h. Ethanol solution (2 mL) of **S11** (0.159 g, 0.60 mmol) was added to the mixture and sodium acetate was added to adjust pH to 5.0. The reaction mixture was stirred at 0℃ for 3 h. H_2_O was added to the mixture and the mixture was extracted with ethyl acetate. The organic phase was washed with H_2_O and brine, dried over anhydrous Na_2_SO_4_ and then concentrated at reduced pressure. The residue was purified by column chromatography to afford the product **Ⅲ-1143** (110 mg, 59.6%) as a red solid. ^1^H NMR (400 MHz, CDCl_3_) δ 7.89 – 7.80 (m, 4H), 7.47 (t, *J* = 7.3 Hz, 2H), 7.37 (t, *J* = 7.3 Hz, 1H), 6.77 (d, *J* = 9.2 Hz, 2H), 4.30 (t, *J* = 6.3 Hz, 2H), 3.64 (t, *J* = 6.3 Hz, 2H), 3.48 (q, *J* = 7.1 Hz, 2H), 2.70 – 2.64 (m, 2H), 2.64 – 2.58 (m, 2H), 1.22 (t, *J* = 7.1 Hz, 3H). ^13^C NMR (101 MHz, CDCl_3_) δ 177.6, 172.1, 153.2, 150.0, 143.8, 129.5, 129.0, 125.2, 122.2, 111.4, 61.9, 48.7, 45.5, 28.8, 12.3. ESI-MS: calcd for C_20_H_22_N_3_O_4_^-^ [M-H]^-^ 368.1616, found 368.1605.

To a solution of 4-phenylazophenol (594 mg, 3.0 mmol) in CH_3_CN (30 mL) was added methyl 6-bromohexanoate (950 µL, 6.0 mmol) and K_2_CO_3_ (2.0 g, 15 mmol). The mixture was gently refluxed for 1 day. Then the mixture was filtered under reduced pressure and the filtrate was poured into H_2_O. The product was extracted with ethyl acetate. The combined organic layer was washed with H_2_O and brine, dried over anhydrous Na_2_SO_4,_ filtered and concentrated under reduced pressure. Then MeOH/THF (1:1, 20 mL) and NaOH (800 mg, 20 mmol) were added to the crude product and the mixture was stirred at room temperature for 2 h. H_2_O was added to the mixture and the mixture was extracted with DCM. The organic phase was washed with H_2_O and brine, dried over anhydrous Na_2_SO_4_ and then concentrated at reduced pressure. The residue was purified by column chromatography to afford the product **Ⅲ-1142** (480 mg, 51.3 %) as a yellow solid. ^1^H NMR (400 MHz, CDCl_3_) δ 8.04 – 7.88 (m, 4H), 7.56 (t, *J* = 7.4 Hz, 2H), 7.49 (t, *J* = 7.4 Hz, 1H), 7.05 (d, *J* = 8.6 Hz, 2H), 4.10 (t, *J* = 7.3 Hz, 2H), 2.47 (t, *J* = 7.3 Hz, 2H), 1.91-1.80 (m, 4H), 1.68 – 1.59 (m, 2H). ^13^C NMR (101 MHz, CDCl_3_) δ 179.8, 161.6, 152.8, 146.9, 130.3, 129.0, 124.8, 122.6, 114.7, 68.0, 34.0, 28.9, 25.6, 24.4. ESI-MS: calcd for C_18_H_19_N_2_O_3_^-^ [M-H]^-^ 311.1401, found 311.1414.

Methyl 4-aminobenzoate (302 mg, 2 mmol) in 5 mL of ethanol was cooled to 0℃. Pre-cooled aqueous solution of potassium nitrite (204 mg, 2.4 mmol) and aqueous solution of sulfuric acid (330 µL, 6.0 mmol) were added to the mixture and stirred at 0°C for 1 h. Ethanol solution (10 mL) of N-ethyl-N-hydroxyethylaniline (396 mg, 2.4 mmol) was added to the mixture and sodium acetate was added to adjust pH to 5.0. The reaction mixture was stirred at 0℃ for 3 h. H_2_O was added to the mixture and the mixture was extracted with ethyl acetate. The organic phase was washed with H_2_O and brine, dried over anhydrous Na_2_SO_4_, filtered and then concentrated at reduced pressure. The residue was purified by column chromatography to afford the product **Ⅲ-1140** (510 mg, 92.1%) as a red solid. ^1^H NMR (400 MHz, DMSO-*d6*) δ 8.10 (d, *J* = 7.6 Hz, 2H), 7.83 (t, *J* = 7.6 Hz, 4H), 6.87 (d, *J* = 8.1 Hz, 2H), 3.69 – 3.59 (m, 2H), 3.59 – 3.46 (m, 4H), 1.17 (t, *J* = 6.3 Hz, 3H). ^13^C NMR (101 MHz, DMSO-*d6*) δ 167.7, 155.5, 151.6, 142.8, 130.9, 126.0, 122.1, 111.7, 58.8, 52.6, 45.6, 12.5. ESI-MS: calcd for C_17_H_18_N_3_O_3_^-^ [M-H]^-^ 312.1354, found 312.1352.

Methyl 4-aminobenzoate (1.51 g, 10 mmol) in 20 mL of ethanol was cooled to 0℃. Pre-cooled aqueous solution of potassium nitrite (1.02 g, 12 mmol) and sulfuric acid (1.6 mL, 30 mmol) were added to the mixture and stirred at 0°C for 1 h. Sulfamic acid (2.4 g, 25 mmol) was added and the reaction was stirred at 0°C for 0.5 h. Ethanol solution (10 mL) of 2,5-dimethoxyaniline (1.84 g, 12 mmol) was added to the mixture and sodium acetate was added to adjust pH to 5.0. The reaction mixture was stirred at 0℃ for 3 h. H_2_O was added to the mixture and the mixture was extracted with ethyl acetate. The organic phase was washed with H_2_O and brine, dried over anhydrous Na_2_SO_4_, filtered and then concentrated at reduced pressure. The residue was purified by column chromatography to afford the product **S12** (2.8 g, 88.9%) as a red solid. ^1^H NMR (400 MHz, DMSO) δ 8.08 (d, *J* = 6.5 Hz, 2H), 7.80 (d, *J* = 6.5 Hz, 2H), 7.31 (d, *J* = 1.7 Hz, 1H), 6.47 (d, *J* = 1.7 Hz, 1H), 6.34 (s, 2H), 3.90 (s, 6H), 3.82 (s, 3H). ^13^C NMR (101 MHz, DMSO) δ 166.4, 156.8, 156.5, 147.0, 141.7, 131.9, 130.8, 128.8, 122.0, 97.3, 96.5, 56.4, 55.9, 52.6. ESI-MS: calcd for C_16_H_18_N_3_O_4_^-^ [M+H]^+^ 316.1292, found 316.1287.

To a pre-cooled solution of above compound **S12** (630 mg, 2.0 mmol) in CH_3_CN was added NOBF_4_ (257 mg, 2.2 mmol). The reaction mixture was stirred at 0°C for 15 min. Then N-ethyl-N-hydroxyethylaniline (396 mg, 2.4 mmol) was added and the mixture was stirred at 0°C for 2 h. H_2_O was added, followed by the addition of aqueous NaOAc (2 M) to adjust pH to 5. The mixture was concentrated at reduced pressure. H_2_O was added and the mixture was extracted with EtOAc. The organic phase was washed with H_2_O and brine, dried over anhydrous Na_2_SO_4_ and then concentrated at reduced pressure. Then MeOH-THF (1:1, 20 mL) and NaOH (800 mg, 20 mmol) was added to the above crude product and the mixture was stirred at room temperature for 24 h. The mixture was concentrated at reduced pressure, followed by the addition of aqueous HOAc (1 M) to adjust pH to 5. The mixture was extracted with ethyl acetate. The organic phase was washed with H_2_O and brine, dried over anhydrous Na_2_SO_4_, filtered and then concentrated at reduced pressure. The residue was purified by column chromatography to afford the product **Ⅲ-1105** (60 mg, 6.3%) as a purple solid. ^1^H NMR (400 MHz, DMSO) δ 8.17 (d, *J* = 7.8 Hz, 2H), 7.97 (d, *J* = 7.8 Hz, 2H), 7.83 (d, *J* = 8.5 Hz, 2H), 7.45 (s, 1H), 7.40 (s, 1H), 6.89 (d, *J* = 8.5 Hz, 2H), 4.02 (s, 3H), 3.97 (s, 3H), 3.65 (t, *J* = 6.3 Hz, 2H), 3.61 – 3.50 (m, 4H), 1.19 (t, *J* = 6.3 Hz, 3H). ^13^C NMR (101 MHz, DMSO) δ 169.9, 155.1, 152.9, 151.6, 150.8, 145.8, 143.7, 141.8, 131.0, 126.2, 123.0, 111.8, 101.0, 100.5, 60.2, 58.8, 56.8, 52.6, 45.7, 12.5. ESI-MS: calcd for C_25_H_26_N_5_O_5_^-^ [M-H]^-^ 476.1939, found 476.1935.

Compound **Ⅲ-1127** was obtained as a red solid based on the same procedure for preparing Compound **Ⅲ-1105**. ^1^H NMR (400 MHz, DMSO) δ 12.29 (s, 1H), 8.07 (d, *J* = 8.5 Hz, 2H), 8.02 – 7.91 (m, 4H), 7.86 (d, *J* = 8.5 Hz, 2H), 7.63 (d, *J* = 7.4 Hz, 3H), 6.90 (d, *J* = 8.9 Hz, 2H), 4.32 – 4.13 (m, 2H), 3.82 – 3.59 (m, 4H), 3.59 – 3.30 (m, 4H), 1.18 (t, *J* = 6.9 Hz, 3H). ^13^C NMR (101 MHz, DMSO) δ 173.8, 172.7, 154.4, 152.6, 152.3, 151.3, 143.3, 132.1, 130.0, 129.6, 126.0, 124.2, 123.3, 123.1, 112.0, 62.0, 48.7, 45.4, 29.2, 29.1, 12.5. ESI-MS: calcd for C_26_H_26_N_5_O_4_^-^ [M-H]^-^ 472.1995, found 472.1990.

## Chemical survivability tests of Raman encoding compounds

## TentaGel S NH_2_ resins (NH_2_ loading: 0.24 mmol/g) were swollen in DMF for 4 h. Each Raman encoding compound was dissolved in a solution of Cl-HOBt (1.2 eq) and DIC (2.0 eq) in DMF to obtain the activated solution. After 10 minutes the solution was added to the beads. The coupling reaction was allowed to proceed at room temperature for 3 h and monitored by Kaiser Test. ^[1, 2]^ After the filtration, the beads were washed with DMF (3 × 1 mL), MeOH (3 × 1 mL) and DMF (3 × 1 mL), respectively. Then, the beads were divided into 3 equal portions. The first portion was treated with 20% piperidine in DMF at room temperature for 2 h. The second portion was treated with 50% TFA in DCM at room temperature for 3 h. The third portion was untreated and used as the control. The treated beads were filtered and washed with DMF, MeOH, DMF and PBS (pH = 7.4), respectively. The obtained beads were measured under the confocal Raman microscope. By comparing the treated beads and untreated beads, the relative Raman intensity of each encoding compound’s characteristic peak to the bead’s polystyrene peak at 1002 cm^-1^ was used to determine the chemical survivability of the encoding compound. Figure S1 represents an example of the chemical-resistant Compound II-2160 and an example of the chemical-unstable Compound II-2156 in TFA treatment. Similar survivability tests were done with the treatments in imidazole/hydroxylamine hydrochloride and aqueous DMSO as well. Then, all the encoding compounds were tested based on this strategy.


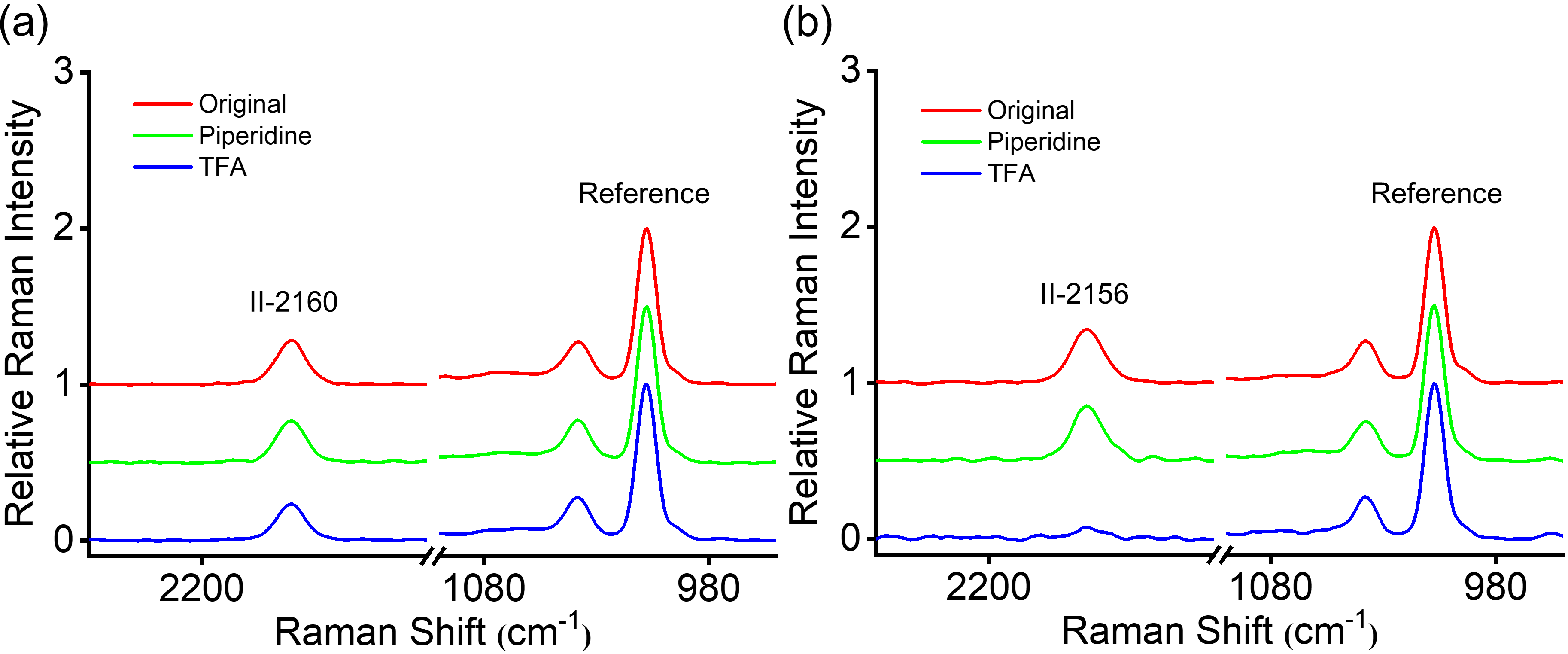


**Figure S1.** Representative Raman spectra of the untreated beads and the beads treated in piperidine and TFA for Compound **II-2160** (a) and **II-2156** (b), respectively.

## Synthesis of decimal Raman codes

The codes were designed as a series of Raman intensities increasing with a fixed ratio of ~1.5× for each of the increasing codes. Compound **I-2245**/**I-2220**, **II-2160**/**II-2110**, and **III-1140**/**III-1105** were combined as the compound pairs in each frequency ranges, respectively. Within the pair, one compound was used for the reference (named as the reference compound) and the second compound was used for the signal (named as the coding compound). **Table S1** lists the designed amount ratios of the coding compound versus the reference compound, which also represent the expected relative Raman intensity of the codes. For example, when no coding compound was added into the synthesis, the coupling the reference compound produced Code #0. When the coding compound’s amount was ~0.2 time of the reference compound’s amount, the synthesis produced Code #1 with the signal peak intensity ~0.2 time of the reference peak. With such a design, we obtained the decimal codes of #0-9. There was ~50% intensity increase for each increasing code, avoiding cross talking between the adjacent codes.

**Table S1.** Design of decimal Raman codes.

| Designed Codes | 0 | 1 | 2 | 3 | 4 | 5 | 6 | 7 | 8 | 9 |
| --- | --- | --- | --- | --- | --- | --- | --- | --- | --- | --- |
| Expected Relative Raman Intensity | 0 | 0.2 | 0.3 | 0.45 | 0.67 | 1.0 | 1.5 | 2.25 | 3.37 | 5.0 |

Experimentally, TentaGel S NH_2_ resins (loading 0.24 mmol/g) were swollen in DMF for 4 h. The reference compound and the coding compound were separately dissolved in a solution of Cl-HOBt (1.2 eq) and DIC (2.0 eq) in DMF. Depending on their relative Raman intensities, the activated solutions were diluted to obtain an equal intensity levels for the reference compound and the coding compound. Then these two stock solutions were mixed with the predesigned volume ratios of 0.0, 0.2, 0.3, 0.45, 0.67, 1.0, 1.5, 2.25, 3.37 and 5.0, respectively. The mixture was added to the TentaGel beads. The coupling reaction was allowed to proceed at room temperature for 3 h and monitored by Kaiser Test. The beads were filtered and washed with DMF, MeOH and DMF, respectively. The Raman spectra of the obtained beads were measured under a confocal Raman microscope.


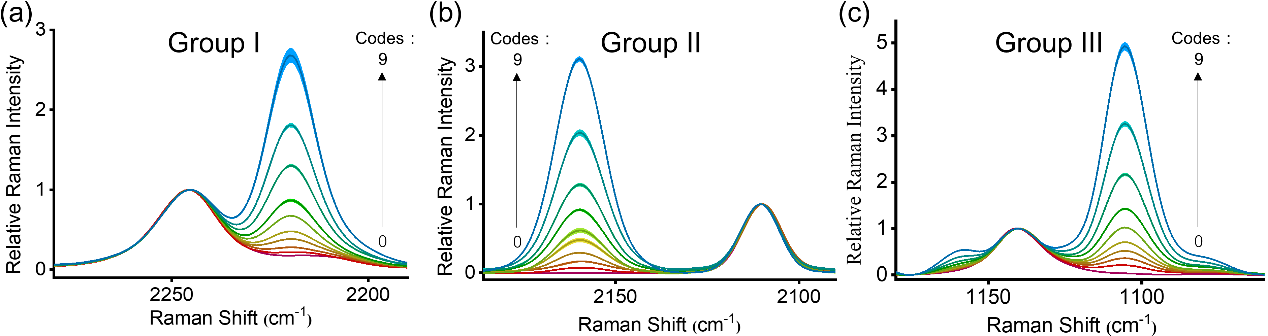


**Figure S2.** Decimal Raman codes in the frequency regions of Group I, II and III, respectively.

## Partial allocation of Dde deprotection


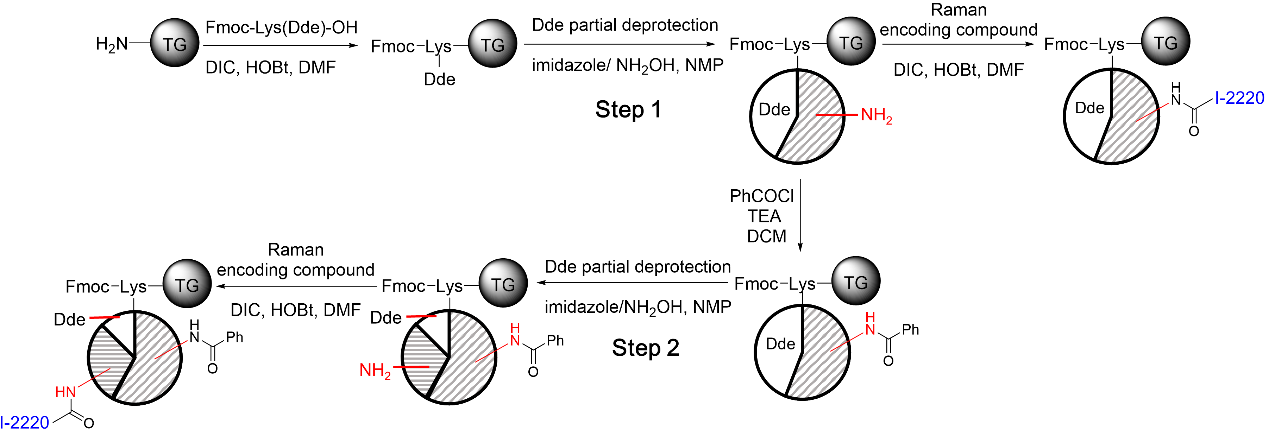


**Figure S3**. Partial allocation of Dde deprotection with the control of deprotection time.

The partial allocation of the side-chain amino groups can be controlled by adjusting the deprotection time in imidazole/NH_2_OH at 25℃. TentaGel S NH_2_ resins (20 mg, loading 0.24 mmol/g) were swollen in DMF for 4 h. 20 mg of Fmoc-Lys(Dde)-OH (0.04 mmol), 8.1 mg of Cl-HOBt (0.048 mmol) and 12 µL of DIC (0.08 mmol) were dissolved in 0.5 mL of DMF to obtain the activated solution. After 10 min of activation the solution was added to the beads. The coupling reaction was allowed to proceed for 3 h at room temperature and monitored by Kaiser Test. After the filtration, the beads were washed with DMF, MeOH and DMF, respectively.

NH_2_OH·HCl (222 mg, 3.2 mmol) and imidazole (163 mg, 2.4 mmol) were dissolved in 10 mL of NMP. The solution was then added to the above Fmoc-Lys (Dde)-TentaGel beads and shaken at 25℃. Small portion of the beads were taken out from the solution every 0.5 h and washed with DMF, MeOH and DMF. An activated solution of Compound **II-2220** (9.8 mg, 0.04 mmol), Cl-HOBt (10 mg, 0.05 mmol) and DIC (12 μL, 0.08 mmol) in 1.0 mL of DMF was added into the above resin beads. The coupling reaction was allowed to proceed at room temperature for 3 h and monitored by Kaiser Test. After the filtration, the beads were washed with DMF, MeOH and DMF, respectively. The Raman spectra of the obtained beads were measured under a confocal Raman microscope.

To test the second proportional Dde deprotection, 60% of the free amino group on the lysine side chain was first blocked by benzoyl chloride and triethylamine in CH_2_Cl_2_ based on the deprotection and coupling procedure described above. Then, the deprotection solution of NH_2_OH/imidazole in NMP was added to the beads and shaken at 25℃. Small portion of the beads were taken out from the solution every 0.5 h and washed with DMF, MeOH and DMF, respectively. The beads were couple with Compound **II-2220** and then measured under a confocal Raman microscope.

## Synthesis of the combinatorial Raman codes


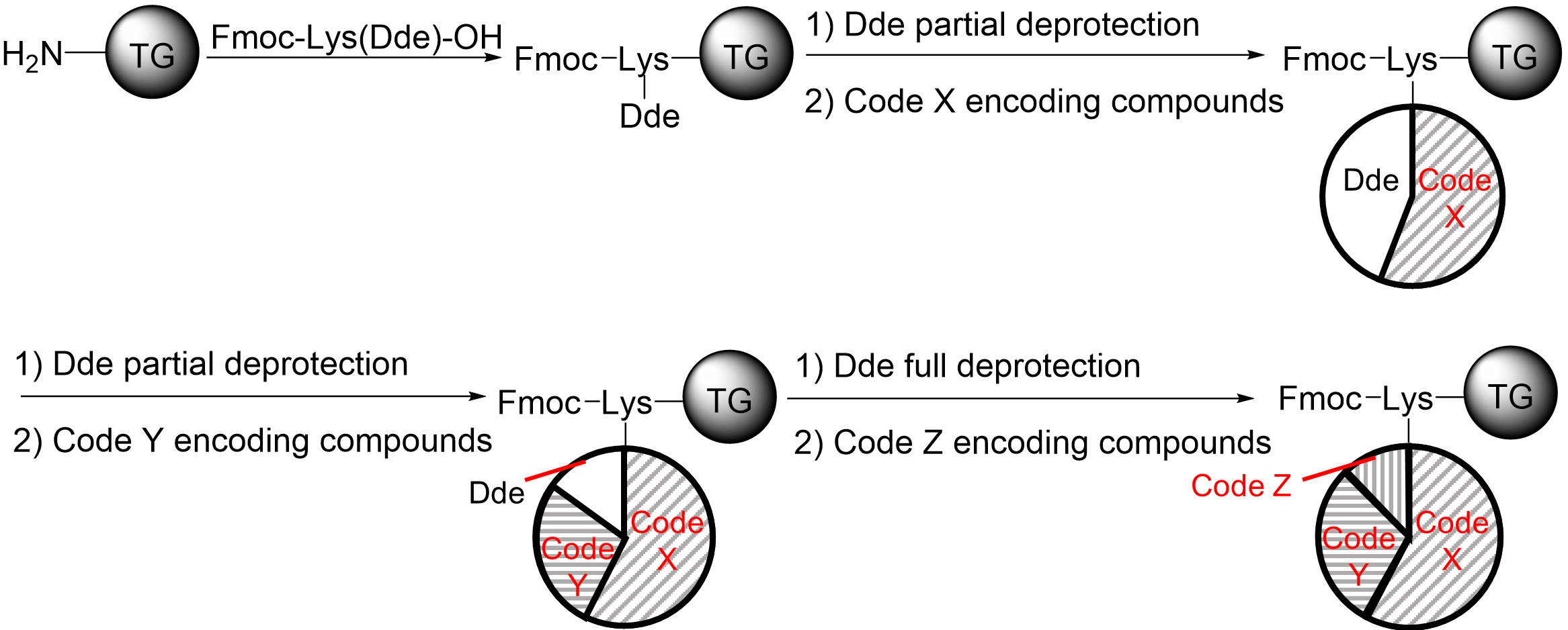


**Figure S4.** Step-by-step Dde deprotection for setting up 3 digit Raman codes.

Fmoc-Lys (Dde)-TentaGel was deprotected by the solution of NH_2_OH·HCl (0.32 M) and imidazole (0.24 M) in NMP. The reaction was allowed to proceed for 1.5 h at 25℃. After the filtration and washing, **Code X** (**II-2160, II-2110**) was constructed using the coupling procedure described above. The activated solution of the coding compound (**II-2160**) and the reference compound **(II-2110**) were mixed by a predetermined ratio of 0.0, 0.2, 0.3, 0.45, 0.67, 1.0, 1.5, 2.25, 3.37 and 5.0, respectively. The coupling reaction was allowed to proceed at room temperature for 3 h and monitored by Kaiser Test. After the reaction was complete, the beads were filtered and washed by DMF, MeOH and DMF, respectively.

The beads were further deprotected by the solution of NH_2_OH·HCl (0.32 M) and imidazole (0.24 M) in NMP for another 1.5 h at 25℃. After the activated solution of the coding compound (**I-2220**) and the reference compound (**I-2245**) were mixed by a predetermined ratio of 0.0, 0.2, 0.3, 0.45, 0.67, 1.0, 1.5, 2.25, 3.37 and 5.0, respectively, the same procedure was taken to construct **Code Y** and obtain Y encoded beads.

The beads were further deprotected by the solution of NH_2_OH·HCl (0.32 M) and imidazole (0.24 M) in NMP for another 3 h at 25℃. Compound **III-1140** presented a small shoulder peak at ~1160 cm^-1^ which might interfere with the code identification (**Figure S2**). Therefore, to construct the combinatorial Raman codes, we used it as the coding compound instead of the reference compound to avoid the potential interference. After the activated solution of the coding compound **III-1140** and the reference compound (**III-1105**) were mixed by a predetermined ratio of 0.0, 0.2, 0.3, 0.45, 0.67, 1.0, 1.5, 2.25, 3.37 and 5.0, respectively, the same procedure was taken to construct **Code Z** and obtain Z encoded beads.

The combinatorial Raman encoded beads were subjected to the chemical stability tests using the same procedure described in the previous section.

**Chemical survivability tests of combinatorial Raman codes**

The Code #5 in each frequency range was used to test the chemical survivability in 20% piperidine in DMF and 50% TFA in DCM based on the same procedure described above. Then, the Raman spectra of the obtained beads were measured under a confocal Raman microscope. The codes’ chemical survivability was determined by comparing the relative Raman intensities of the coding compound versus the reference compound for the treated and untreated beads.

## Synthesis of the combinatorial Raman encoded peptide library

**
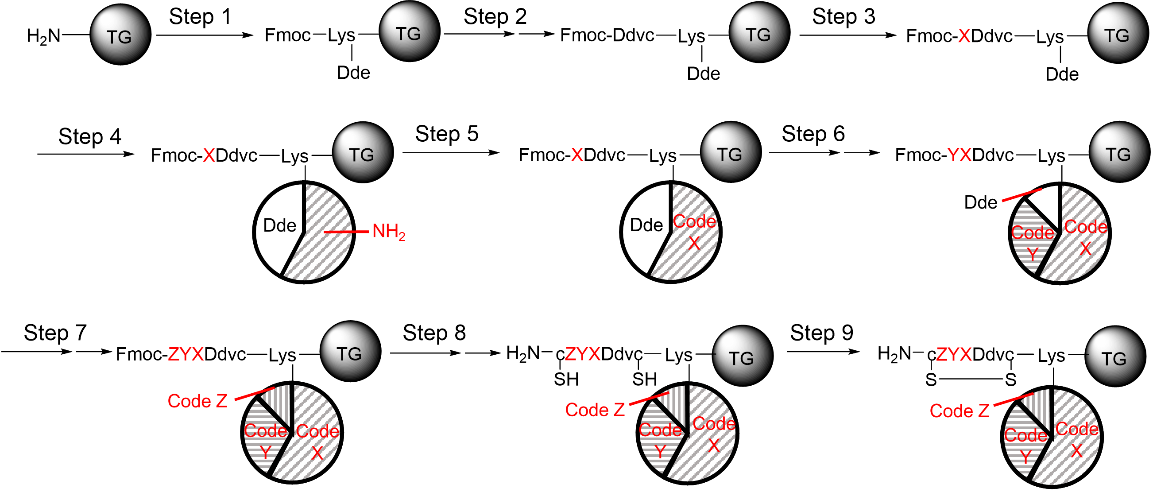
**

**Figure S5.** Split and pool synthesis procedure of Raman-encoded peptide library

TentaGel S NH_2_ resins (400 mg, loading 0.24 mmol/g) were swollen in DMF (5 mL) for 4 h. Fmoc-L-Lys (Dde)-OH (160 mg, 0.30 mmol) was dissolved in a solution of Cl-HOBt (61 mg 0.36 mmol) and DIC (93 µL, 0.6 mmol) in DMF. The solution was shaken for 10 min to get activated. Then the solution was added to the beads. The coupling reaction was allowed to proceed at room temperature for 4 h and monitored by Kaiser Test. After the filtration, the beads were washed with DMF, MeOH and DMF, respectively, to obtain Fmoc-Lys(Dde)-TG resin beads.

To the above Fmoc-Lys(Dde)-TG resin beads were added 20% piperidine in DMF. The mixture was shaken for 5 min. After the filtration, the same reagent was added to the beads and the mixture was shaken for additional 15 min at room temperature. Then the beads were washed with DMF, MeOH and DMF, respectively. Fmoc-D-Cys(Trt)-OH (176 mg, 0.3 mmol) was dissolved in a solution of Cl-HOBt (61 mg 0.36 mmol) and DIC (93 µL, 0.60 mmol) in DMF. The solution was shaken for 10 min and then added to the beads. The coupling reaction was allowed to proceed for 4 h at room temperature and monitored by Kaiser Test. After the filtration, the beads were washed with DMF, MeOH and DMF, respectively. Fmoc-D-Val-OH, Fmoc-D-Asp(OtBu)-OH and Fmoc-L-Asp(OtBu)-OH were then coupled to the resin beads step by step based on the same procedure described above to produce Fmoc-Ddvc-Lys (Dde)-TG resin beads.

The above Fmoc-Ddvc-Lys(Dde)-TG resins (150 mg) were first deprotected by 20% piperidine in DMF twice for 5 min and 15min, respectively. Then the beads were washed with DMF, MeOH and DMF, respectively. They were split into 10 equal portions. Activated solutions of 0.03 mmol of the 10 chiral amino acids, Fmoc-L-Ser (*t*Bu)-OH, Fmoc-L-Met-OH, Fmoc-L-Gln(Trt)-OH, Fmoc-D-Asp(O*t*Bu)-OH, Fmoc-L-Phe-OH, Fmoc-L-Gly-OH, Fmoc-L-Asp(O*t*Bu)-OH, Fmoc-L-Arg(Pbf)-OH, Fmoc-D-Phe-OH and Fmoc-D-Val-OH, were prepared by the same procedure as described above. To each portion of the beads, the above 10 activated solution was added. The coupling reaction was allowed to proceed at room temperature for 4 h and monitored by Kaiser Test. After the filtration, the beads were washed with DMF, MeOH and DMF, respectively.


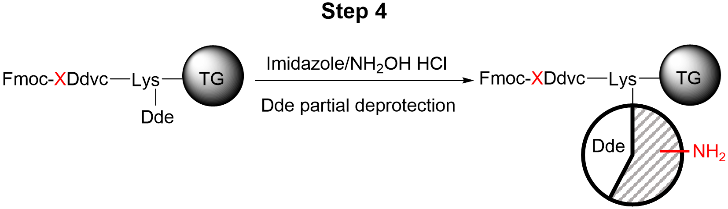


The resin beads were treated with hydroxylamine hydrochloride (444 mg, 6.4 mmol) and imidazole (326 mg, 4.8 mmol) in NMP (20 mL) in the heating oven at 25℃ for 1.5 h. After the filtration, the beads were washed with DMF, MeOH and DMF, respectively.


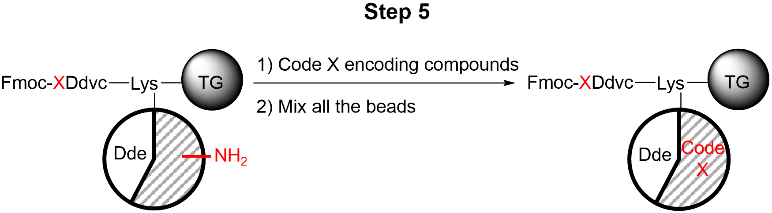


Ten codes of Code X were constructed onto the 10 portions of resin beads using Compound **II-2160** and **II-2110** based on the same procedure of the encoding steps described above. Then, the 10 portions of resin beads were mixed together.


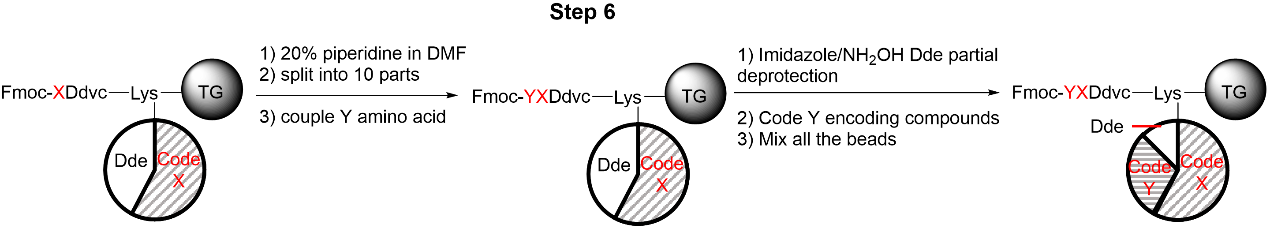


The addition of another 10 amino acids and the corresponding Code Y were constructed based on the same procedure of **Step 3** (coupling 10 different amino acids), **Step 4** (partial deprotection of Dde protecting group, 30%) and **Step 5** (coupling Code Y using Compound **I-2245** and **I-2220**).


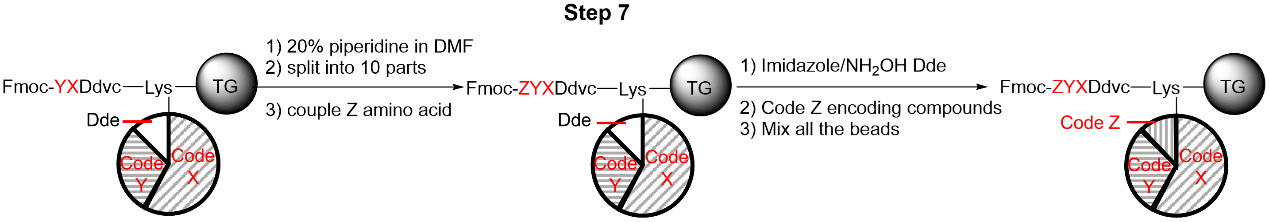


The addition of another 10 amino acids and the corresponding Code Z were constructed based on the same procedure of **Step 3** (coupling 10 different amino acids), **Step 4** (partial deprotection of Dde protecting group, 30%) and **Step 5** (coupling Code Z using Compound **III-1140** and compound **II-1105**).


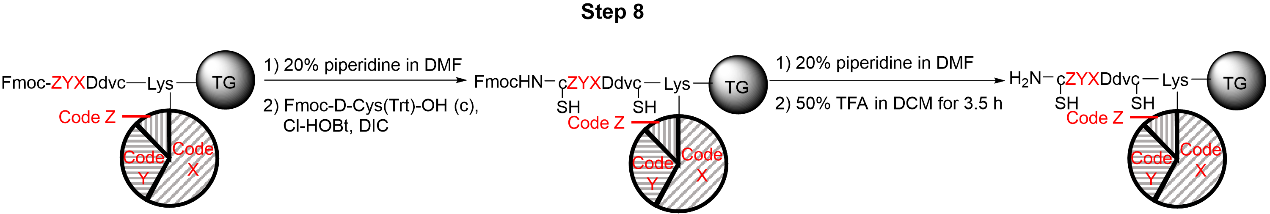


Fmoc-D-Cys (Trt)-OH was coupled to the resin beads based on the same procedure in **Step 2** to obtain linear 8-mer peptides. After the removal of Fmoc, the beads were washed with DMF, MeOH and DMF, respectively. Then a cleavage cocktail solution of 50% TFA in DCM containing 1% triisopropylsilane was added and the reaction was shaken at room temperature for 3.5 h. After the neutralization with 2% DIEA in DCM for twice, the resin beads were washed with MeOH, DCM, DMF: H_2_O (v: v = 6: 4) and water, respectively.


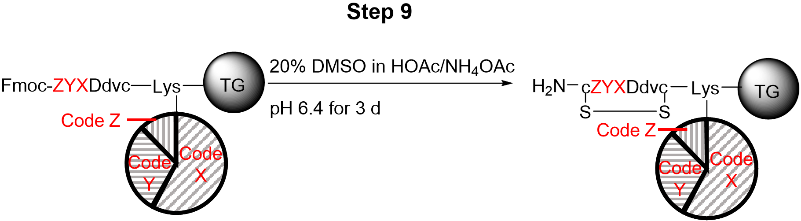


The beads were transferred to a 10 mL reaction vessel, to which was added 5 mL mixture of HOAc/NH_4_OAc buffer (pH=6.4) and DMSO (v: v = 4: 1). The reaction mixture was shaken at room temperature for 3 days. After the reaction was complete, the beads were filtered and washed with PBS (pH=7.4) twice. The obtained encoded OBOC library was stored in 75% EtOH at -20℃ before the screening experiment.

During the synthesis of the coding library, we collected a very small portion of beads right after the exact step when each code was just fabricated. They were measured to provide the calibration of the standard codes. Then after the library was screened, the spectra of the positive hits were compared with the standards to decode the peptide sequences. **Figure S6** below shows the normalized Raman spectra of the standard codes of the library.


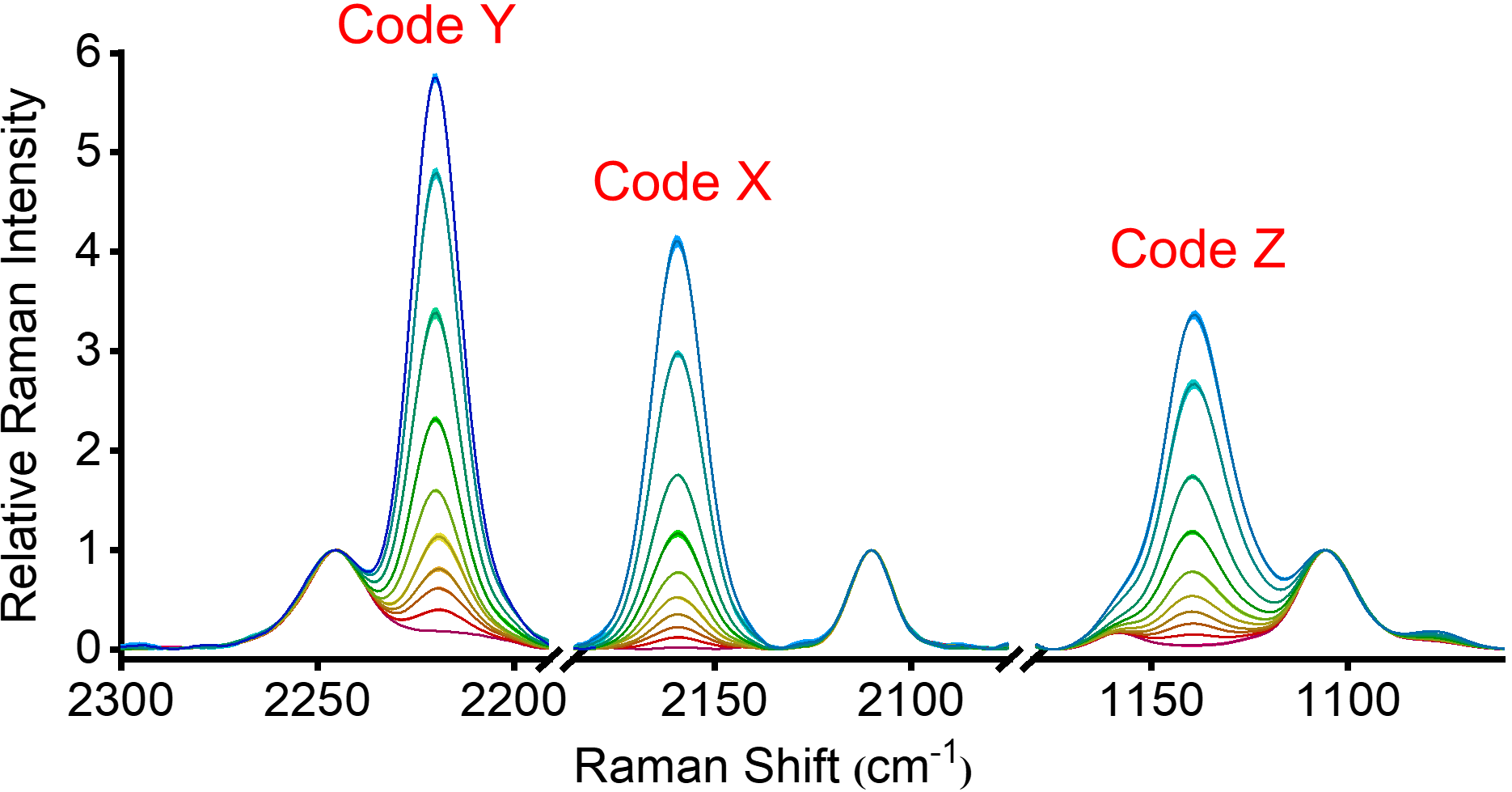


**Figure S6**. Normalized Raman spectra of the standard library codes with the error bars.

References:

1. Tang, Y.; He, C.; Zheng, X.; Chen, X.; Gao, T. Super-capacity information-carrying systems encoded with spontaneous Raman scattering. *Chem. Sci.* **2020**, *11*, 3096-3103.

2. Tang,Y; Thillier, Y.; Liu, R.; Li, X.; Lam, K. S.; **Gao, T.** Single-Bead Quantification of Peptide Loading Distribution for One-Bead One-Compound Library Synthesis Using Confocal Raman Spectroscopy. Anal. Chem. **2017**, 89, 7000-7008.
